# Cocaine preference and neuroadaptations are maintained by astrocytic NMDA receptors in the nucleus accumbens

**DOI:** 10.1126/sciadv.abo6574

**Published:** 2022-07-22

**Authors:** Gajanan P. Shelkar, Pauravi J. Gandhi, Jinxu Liu, Shashank M. Dravid

**Affiliations:** Department of Pharmacology and Neuroscience, Creighton University School of Medicine, 2500 California Plaza, Omaha, NE, USA.

## Abstract

Cocaine-associated memories induce cravings and interfere with the ability of users to cease cocaine use. Reducing the strength of cue-drug memories by facilitating extinction may have therapeutic value for the treatment of cocaine addiction. Here, we demonstrate the expression of GluN1/2A/2C NMDA receptor currents in astrocytes in the nucleus accumbens core. Selective ablation of GluN1 subunit from astrocytes in the nucleus accumbens enhanced extinction of cocaine preference memory but did not affect cocaine conditioning or reinstatement. Repeated cocaine exposure up-regulated GluN2C subunit expression and increased astrocytic NMDA receptor currents. Furthermore, intra-accumbal inhibition of GluN2C/2D-containing receptors and GluN2C subunit deletion facilitated extinction of cocaine memory. Cocaine-induced neuroadaptations including dendritic spine maturation and AMPA receptor recruitment were absent in GluN2C knockout mice. Impaired retention of cocaine preference memory in GluN2C knockout mice was restored by exogenous administration of recombinant glypican 4. Together, these results identify a previously unknown astrocytic GluN2C-containing NMDA receptor mechanism underlying maintenance of cocaine preference memory.

## Teaser

Astrocytic NMDA receptors are potential therapeutic targets for the extinction of cocaine memories.

## INTRODUCTION

Progression from reward to debilitating addiction is a multifaceted and interlinked behavioral sequence that strengthens over the period of drug use, engaging several brain regions, cell types, and signaling pathways. When a drug is paired with environmental stimuli (cues), a motivational memory is formed, predisposing individuals with substance use disorder to relapse after drug abstinence (*1*). Reducing the strength of cue-drug memories could decrease cravings and relapse likelihood. This can be accomplished by facilitating extinction learning and/or disrupting drug-cue association memories (*2*, *3*). Extinction involves inhibitory learning by pairing an unreinforced stimulus to the initial drug-paired cues (*2*, *4*).

Although neuronal mesocorticolimbic dopamine circuitry is thought to be the primary substrate underlying drug addiction, a role of astrocytes in drug abuse has recently been identified (*5*–*7*). Recent studies have established that astrocytes participate in synaptic transmission, synaptic plasticity, and synapse formation, maintenance, and elimination (*8*). The role of astrocytes has also been identified in neuroplasticity in reward behavior. Specifically, reduced expression of glial cysteine-glutamate exchanger has been found to underlie down-regulated glutamatergic neurotransmission after cocaine exposure (*5*, *7*). In addition, activation of glial Gq-coupled signaling in the nucleus accumbens (NAc) decreases cue-induced cocaine seeking (*9*). Cocaine self-administration and extinction in rodents also produce morphological changes in astrocytes and alter contacts of astrocytic processes with synapses (*10*). Furthermore, cocaine induces neuroadaptations such as the generation of silent synapses, spine maturation, and an increase in synaptic strength (*11*–*13*), and it is conceivable that astrocytes may play a role in these neuroplasticity events upon cocaine experience.

In addition to a variety of receptors, astrocytes have also been found to express *N*-methyl-d-aspartate (NMDA) receptors. Specifically, cortical astrocytes show NMDA receptor currents that have properties distinct from neuronal NMDA receptor currents (*14*, *15*). They appear to be mainly mediated by GluN2C/2D-containing receptors because of their low sensitivity to Mg^2+^ block and inhibition by GluN2C/2D-selective blockers (*15*). In addition, using a GluN2C reporter model and fluorescence in situ hybridization assays, we recently demonstrated the unique expression of GluN2C in astrocytes in several corticolimbic regions, including the striatum (*16*, *17*). Despite accumulating evidence for the expression of astrocytic NMDA receptors, its role in regulating neuronal plasticity and behavior is unknown.

We investigated the potential role of astrocytic NMDA receptors in drug-induced behavior and plasticity. We found that astrocytic NMDA receptors are critical for maintaining cocaine preference memory, and ablation of NMDA receptors from NAc astrocytes facilitates the extinction of cocaine-induced conditioned place preference (CPP). Furthermore, we show that astrocytic NMDA receptors in the NAc core are composed of GluN2C subunits. Cocaine conditioning increases NAc GluN2C expression, which is critical for the maintenance of cocaine memory, as evidenced by enhanced extinction in GluN2C knockout (KO) mice. Molecular studies demonstrated that astrocytic NMDA receptors and GluN2C subunits are critical for cocaine-induced dendritic spine and synaptic plasticity by modulating synaptogenic factor expression. Together, these studies identify a previously unknown astrocytic NMDA receptor mechanism for the maintenance of cocaine memory.

## RESULTS

### GluN2C subunit–containing NMDA receptors in NAc are the predominant NMDA receptors expressed in astrocytes

**Fig. 1.**
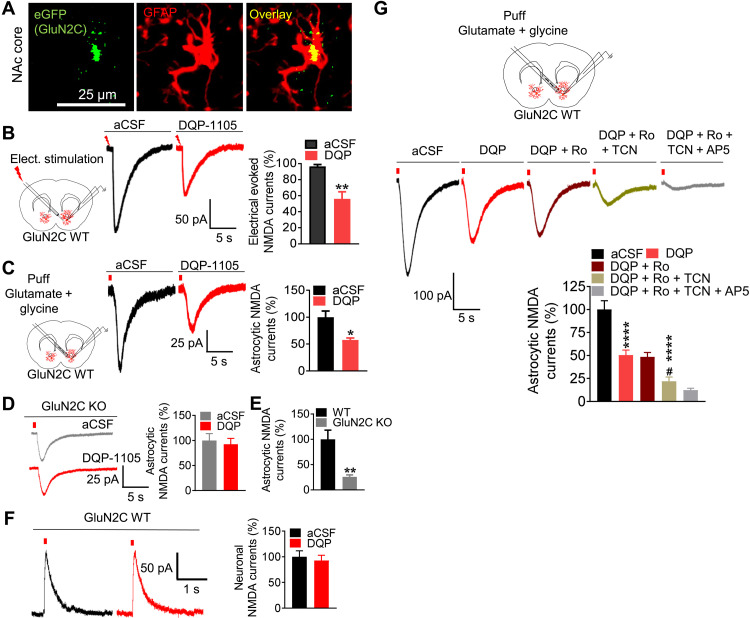
Astrocytes, but not neurons, in the NAc core express GluN2C-containing NMDA receptors with a GluN1/GluN2A/GluN2C stoichiometry. (**A**) Immunohistochemical analysis of NAc core sections for GFAP and eGFP (GluN2C) in the reporter model. Colocalization of eGFP with GFAP demonstrates the expression of the GluN2C subunit in astrocytes. (**B** and **C**) Experimental strategy for whole-cell voltage-clamp recordings from astrocytes (holding potential of −70 mV). Electrical stimulation (B) or puff application of glutamate + glycine (C) elicited NMDA receptor currents in SR101-labeled WT astrocytes, which were significantly blocked by DQP-1105 (*P = 0.0395 and **P = 0.0082 versus respective aCSF control, paired *t* test; *n* = 5). (**D**) Recordings from NAc astrocytes in GluN2C KO mice. DQP-1105 failed to reduce NMDA currents in GluN2C KO mice. (**E**) Bar graph showing differences in astrocytic NMDA receptor currents in WT and GluN2C KO mice. Significantly smaller amplitude of the astrocytic NMDA receptor currents in GluN2C KO [gray trace in (D)] mice than WT [black trace in (C)] (**P = 0.0034, unpaired *t* test; *n* = 7 to 9). (**F**) Whole-cell voltage-clamp recordings from NAc MSNs in WT mice (holding potential of +40 mV). DQP-1105 did not produce any significant reduction in neuronal NMDA receptor currents (*P* > 0.05, paired *t* test; *n* = 8). (**G**) Electrophysiological characterization of NMDA receptor composition in astrocytes using selective pharmacological tools. Puff application of glutamate + glycine elicited NMDA receptor currents in astrocytes in the NAc core. Approximately 50% of this current was sensitive to GluN2C/2D antagonist, DQP-1105 (****P = 0.0001). GluN2B-selective antagonist Ro25-6981 did not produce any significant inhibition of the current. GluN2A-selective antagonist TCN201 produced a further reduction in the current (****P < 0.0001 versus aCSF, ^#^*P* < 0.0189 versus DQP-1105 treatment). One-way analysis of variance (ANOVA) showed significant effect of drug treatments on NMDA receptor currents (*F*_4,35_ = 28, *P* < 0001; *n* = 6 to 9).

We and others have previously demonstrated the unique expression of GluN2C subunit in astrocytes in some corticolimbic regions (*16*–*20*). Here, we first examined the cell type–specific expression of GluN2C in the NAc core and prefrontal cortex (PFC) using an enhanced green fluorescent protein (eGFP) reporter model that we have previously described (*16*). Similar to our observations in the striatum, GluN2C was found to colocalize with glial fibrillary acidic protein (GFAP)–expressing astrocytes but not neurons or microglia in the NAc core (Fig. 1A and fig. S1, A to D) and PFC (fig. S1, E to H). Previous studies have demonstrated that astrocytic NMDA receptor currents have different subunit contribution than neuronal NMDA receptors. Specifically, NMDA receptors in cortical astrocytes appear to have GluN2C/2D subunit contribution (*14*, *15*). By performing whole-cell patch-clamp recordings in astrocytes in the NAc core, we examined the subunit composition of NMDA receptor currents using pharmacological tools. We found that electrical stimulation (Fig. 1B) or puff application of glutamate + glycine (Fig. 1C) elicited NMDA receptor currents in SR101-labeled astrocytes in wild-type (WT) mice. The NMDA receptor currents were significantly reduced by bath application of GluN2C/2D-selective antagonist DQP-1105 (*P* = 0.0082, Fig. 1B; *P* = 0.0395, Fig. 1C; paired *t* test). However, DQP-1105 failed to inhibit astrocytic NMDA receptor currents in GluN2C KO mice (Fig. 1D). In GluN2C KO mice, the astrocytic NMDA receptor currents were significantly smaller in amplitude than in WT (*P* = 0.0034, unpaired *t* test; Fig. 1E). This set of data directly demonstrates the contribution of GluN2C subunit to NMDA receptor currents in astrocytes. Furthermore, NMDA receptor currents from medium spiny neurons (MSNs) in WT mice were not inhibited by DQP-1105 (Fig. 1F), demonstrating the selective expression of GluN2C-containing NMDA receptors in astrocytes and lack thereof in neurons. We also examined the contribution of other subunits in NMDA receptor currents in astrocytes using GluN2A- and GluN2B-selective agents. Bath application of GluN2A-selective antagonist TCN201 produced ~25% reduction in currents [*P* < 0.0001 versus artificial cerebrospinal fluid (aCSF), *P* < 0.0189 versus DQP-1105 treatment; Fig. 1G], but GluN2B antagonist Ro25-6981 did not produce any inhibition of current. Together, these results demonstrate the presence of NMDA receptor currents in striatal astrocytes and suggest their stoichiometry to be GluN1/2A/2C triheteromers.

### Astrocytic NMDA receptors stabilize cocaine preference memory during extinction

**Fig. 2.**
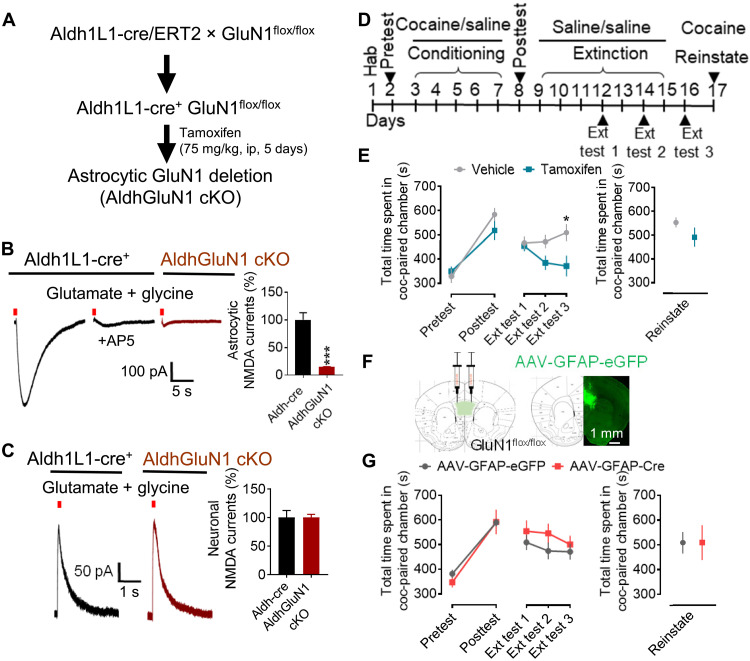
Astrocytic NMDA receptors regulate extinction of cocaine preference. (**A**) Strategy used for conditional deletion of astrocytic NMDA receptors using Aldh1L1-Cre/ERT2 line for tamoxifen-inducible cre recombinase expression in astrocytes. ip, intraperitoneally. (**B** and **C**) Confirmation of astrocytic NMDA receptor deletion. (B) Whole-cell voltage-clamp recordings from NAc core astrocytes conducted in Aldh1L1-cre (WT)– and AldhGluN1-deleted (AldhGluN1 cKO) mice. Puff application of glutamate (1 mM) + glycine (100 μM) produced AP5-sensitive currents in Aldh1L1-cre mice. Negligible currents were seen in AldhGluN1-deleted mice (***P = 0.0005, unpaired *t* test; *n* = 7 to 10). (C) No change in neuronal NMDA currents in Aldh1L1-cre compared to AldhGluN1-deleted mice (*P* > 0.9999, unpaired *t* test; *n* = 10 each). (**D**) Experimental approach used for cocaine CPP experiments. Hab, habituation; Ext, extinction. (**E**) Effect of astrocytic NMDA receptor deletion on cocaine CPP. Cocaine conditioning increased time spent in the cocaine-paired chamber in vehicle- and tamoxifen-treated mice to a similar extent. Conditional deletion of astrocytic NMDA receptors facilitated extinction with reduced time spent in the cocaine-paired chamber (*P = 0.0105). Two-way ANOVA showed significant effect of genotype on extinction (*F*_1,13_ = 6.504, *P* = 0.0242; *n* = 7 to 8). No significant difference in reinstatement between vehicle- and tamoxifen-treated groups (*P* = 0.2079, unpaired *t* test). (**F**) Strategy for local ablation of astrocytic NMDA receptors from PFC. Schematic showing expression and site of virus injection. Coronal image from GluN1^flox/flox^ mice injected with AAV-GFAP-eGFP showing virus expression in PFC. (**G**) GluN1^flox/flox^ mice injected with AAV-GFAP-eGFP (*n* = 7) or AAV-GFAP-mCherry-cre (*n* = 6) in PFC were tested in CPP. No significant difference was observed in two groups following cocaine conditioning and extinction (*P* > 0.9999 each, two-way ANOVA) and reinstatement (*P* = 0.449, unpaired *t* test). No significant effect of treatment (cre or control) on extinction was observed (*F*_1,11_ = 2.707, *P* = 0.1282, two-way ANOVA).

We next addressed the role of astrocytic NMDA receptors in cocaine preference memory using a genetic model. NMDA receptors are heteromeric complexes composed of two GluN1 subunits and two GluN2 and/or GluN3 subunits. GluN1 is an obligatory subunit, and its deletion should result in an overall loss of NMDA receptor function. We used the Aldh1L1-CreERT2 mouse line together with GluN1^flox/flox^ mice to achieve deletion of the GluN1 subunit from astrocytes (Fig. 2A). Cre expression was induced by tamoxifen injection. To validate loss of NMDA receptor function from astrocytes, we recorded NMDA receptor currents from SR101-labeled NAc core astrocytes in acute brain slices from Aldh1L1-CreERT2 (WT) and astrocytic GluN1-deleted mice [AldhGluN1 conditional KO (cKO)] (Fig. 2B). In WT mice, puff application of glutamate + glycine elicited NMDA receptor currents, which were inhibited by AP5 (NMDA receptor antagonist), demonstrating that these were NMDA receptor currents. In contrast, recordings from astrocytes in brain slices from astrocytic GluN1-deleted mice showed negligible currents (*P* = 0.0005 versus WT, unpaired *t* test) following puff application of glutamate + glycine, suggesting loss of function of NMDA receptors in astrocytes. We also performed recordings from neurons in brain slices from WT and astrocytic GluN1-deleted mice (Fig. 2C) and did not observe any significant differences in the NMDA receptor currents (*P* > 0.9999, unpaired *t* test). In addition, down-regulation of GluN1 subunits in the tamoxifen injected group was confirmed in PFC and NAc using immunohistochemistry and immunoblotting. Significant reduction in GluN1 puncta was observed in both regions (PFC: *P* < 0.0001, NAc core: *P* = 0.0483, unpaired *t* test; fig. S2A). In addition, a reduction in protein expression was observed in immunoblotting experiments (PFC: *P* = 0.05, NAc: *P* = 0.0478, unpaired *t* test; fig. S2B). These results confirm the specific deletion of NMDA receptors from astrocytes but not neurons.

We next examined cocaine conditioning, extinction, and reinstatement in the CPP paradigm in mice with deletion of GluN1 from astrocytes (Fig. 2D). After cocaine conditioning, mice showed a significant increase in time spent in a cocaine-paired chamber (Fig. 2E). This increase was observed in both control (vehicle-injected) and astrocytic GluN1-deleted (tamoxifen-injected) groups to a similar extent (*P* = 0.2524). During extinction training, saline was paired with both sides of the CPP apparatus, and time spent in the cocaine-paired chamber was examined at three time points. Significant reduction in time spent in the cocaine-paired chamber was observed during the extinction posttest (average across all posttests) in both vehicle group [*P* = 0.0175, one-way analysis of variance (ANOVA)] and tamoxifen-treated mice (*P* = 0.0105, two-way ANOVA), demonstrating the effectiveness of extinction training (fig. S2, C and D). A significantly lower time spent in the cocaine-paired chamber was found in mice with astrocytic GluN1 deletion at extinction test 3 (*P* = 0.0105, two-way ANOVA; Fig. 2E), suggesting that astrocytic NMDA receptors are necessary for retention of cocaine preference memory. No significant difference in cocaine-induced reinstatement was observed between the two groups (*P* = 0.2079, unpaired *t* test; Fig. 2E). Under similar CPP design when saline was used instead of cocaine, no preference for the chambers was observed after conditioning (WT: pretest 350.2 ± 34.03 versus posttest 381 ± 54.06; astrocytic GluN1 deletion: pretest 320.4 ± 32.93 versus posttest 371.8 ± 54.89).

Previous studies have shown expression of NMDA receptor currents in cortical astrocytes (*14*, *15*), which are dependent on GluN2C subunit (*16*). The medial PFC (mPFC) sends projections to the NAc, which play an important role in extinction (*21*, *22*). Thus, we tested whether astrocytic NMDA receptors in the mPFC are important for retention of cocaine preference memory. Local ablation of the GluN1 subunit was achieved by AAV-GFAP-mCherry-cre (AAV-cre) injection into the mPFC (both prelimbic and infralimbic cortex) of GluN1^flox/flox^ mice (Fig. 2F and fig. S2, F and G). AAV-GFAP-eGFP (AAV-control)–injected animals were used as controls. After 4 weeks for sufficient expression and conditional deletion, mice were tested in the cocaine CPP paradigm (Fig. 2D). After conditioning, both AAV-cre– and AAV-control–injected mice showed a similar level of preference to the cocaine-paired chamber (*P* > 0.9999; Fig. 2G and fig. S2E). In addition, no statistically significant differences were observed in posttests after extinction (*P* > 0.9999 at all extinction tests, two-way ANOVA) and reinstatement (*P* = 0.449, unpaired *t* test; Fig. 2G) in the two groups. Thus, the function of astrocytic NMDA receptors in the mPFC may not contribute to the retention of cocaine preference memory during the extinction phase.

### Ablation of NMDA receptors from NAc astrocytes impairs cocaine memory retention

**Fig. 3.**
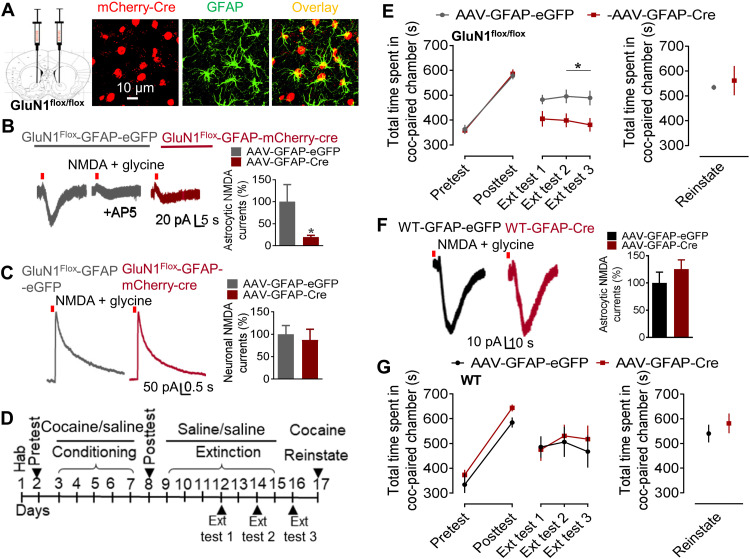
Accumbal astrocytic NMDA receptors are crucial for retention of cocaine place preference memory. (**A**) Schematic showing site of virus injection into the NAc core. Selectivity of AAV-Cre expression to GFAP-positive astrocytes. (**B**) Whole-cell voltage-clamp recordings from NAc core astrocytes conducted in AAV-control– and AAV-Cre–injected GluN1^flox/flox^ mice. Puff application of NMDA (1 mM) + glycine (100 μM) produced AP5-sensitive currents in AAV-control mice. Negligible currents were seen in AAV-cre–injected mice (*P = 0.0129, unpaired *t* test; *n* = 5 to 8). (**C**) No change in neuronal NMDA currents in AAV-cre–injected animals compared to AAV-control (*P* = 0.6851, unpaired *t* test; *n* = 4 to 5). (**D**) Experimental approach implemented for cocaine CPP experiments. (**E**) GluN1^flox/flox^ mice injected with AAV-control or AAV-Cre into the NAc core were tested for cocaine CPP. No difference in the two groups was observed in conditioning (*P* > 0.9999, two-way ANOVA). Astrocyte-specific deletion of NMDA receptors from NAc core accelerates extinction with lower time spent in the cocaine-paired chamber (*P = 0.0459 at extinction test 2 and *P = 0.0198 at extinction test 3). Two-way ANOVA showed significant effect of treatment on extinction (*F*_1,25_ = 7.997, *P* = 0.0091; *n* = 13 to 14). No significant difference was observed in the two groups following reinstatement (*P* = 0.6592, unpaired *t* test). (**F**) Whole-cell voltage-clamp recordings from NAc core astrocytes conducted in AAV-control– and AAV-cre–injected WT (no floxed gene) mice. Application of NMDA + glycine produced similar currents in both groups, suggesting no loss of function (*P* = 0.3598, unpaired *t* test). (**G**) WT mice injected with AAV-control or AAV-cre into the NAc core were tested for cocaine CPP. No difference was observed in the two groups after conditioning (*P* = 0.2668, two-way ANOVA), after extinction (*F*_1,9_ = 0.1175, *P* = 0.7396, two-way ANOVA; *n* = 5 to 6), or after reinstatement (*P* = 0.4652, unpaired *t* test).

Because NMDA receptors in mPFC astrocytes were found not to control cocaine preference, we examined the NAc core as a potential site for this effect. For these experiments, we first conducted recordings from brain slices from mice with a cKO strategy to confirm model specificity of loss of NMDA receptor function. We injected GluN1^flox/flox^ mice with AAV-control or AAV-cre in the NAc core (Fig. 3A and fig. S3A). Following 4 weeks of expression period, whole-cell patch-clamp recordings were conducted from labeled NAc core astrocytes. In AAV-control–injected animals, application of NMDA + glycine onto labeled astrocytes produced currents that were inhibited by AP5 (NMDA receptor antagonist), demonstrating that these were NMDA receptor–specific currents (Fig. 3B). In contrast, recordings from astrocytes in brain slices from AAV-cre–injected animals showed negligible currents following application of NMDA + glycine compared to AAV-control (*P* = 0.0129, unpaired *t* test), suggesting loss of function of astrocytic NMDA receptors. We did not find any change in the evoked NMDA receptor currents when recordings were made from neurons in the vicinity of labeled astrocytes in the AAV-control or AAV-cre groups (*P* = 0.6851, unpaired *t* test), confirming that this manipulation did not affect neuronal NMDA receptor expression (Fig. 3C). We confirmed the cell-specific expression of AAV by immunohistochemistry. AAV-mCherry-cre was found to colocalize with GFAP (astrocytic marker; Fig. 3A) but not with NeuN (neuronal marker), confirming the AAV’s specificity (fig. S3B).

We next sought to understand the specific role of astrocytic NMDA receptors in the NAc core in cocaine preference using the CPP paradigm (Fig. 3D). After cocaine conditioning, both AAV-control and AAV-cre groups showed similar time spent in the cocaine-paired chamber during the posttest (Fig. 3E). A significant reduction in the time spent in the cocaine-paired chamber was observed after extinction training in both groups (average of extinction posttests, AAV-control, *P* = 0.0023; AAV-cre, *P* < 0.0001 versus respective conditioning group, two-way ANOVA; fig. S3, C and D). During the extinction phase, the AAV-cre–injected mice spent significantly less time in the cocaine-paired chamber compared to AAV-control mice, suggesting enhanced extinction upon loss of astrocytic NMDA receptors in the NAc core (*P* = 0.0459 and *P* = 0.0198 at extinction tests 2 and 3, respectively, two-way ANOVA). Both groups reinstated to levels similar to those before extinction sessions, and there was no significant difference between the two groups in the time spent in the cocaine-paired chamber after reinstatement (*P* = 0.6592, unpaired *t* test). To rule out the possibility of any nonspecific effects of cre expression on behavior, we conducted control experiments in WT mice using the same in vivo manipulations (Fig. 3, F and G, and fig. S3E). WT mice (with no floxed gene) were injected with AAV-cre or AAV-control virus into the NAc core. A whole-cell patch-clamp recording from astrocytes of AAV-cre– or AAV-control–injected mice showed similar currents following application of NMDA + glycine, suggesting no loss of function of NMDA receptors (*P* = 0.3598, unpaired *t* test; Fig. 3F). In addition, no significant differences were observed in WT mice injected with AAV-cre or AAV-control in conditioning (*P* = 0.2668, two-way ANOVA), extinction (*P* > 0.9999, two-way ANOVA), or reinstatement (*P* = 0.4652, unpaired *t* test; Fig. 3G). These results demonstrate a lack of nonspecific effects of cre recombinase expression. Together, these results suggest a critical role of astrocytic NMDA receptors in the NAc core in stabilizing cocaine memory but not in cocaine conditioning or reinstatement.

### Repeated cocaine exposure increases GluN2C subunit expression and astrocytic NMDA receptor currents in the NAc

**Fig. 4.**
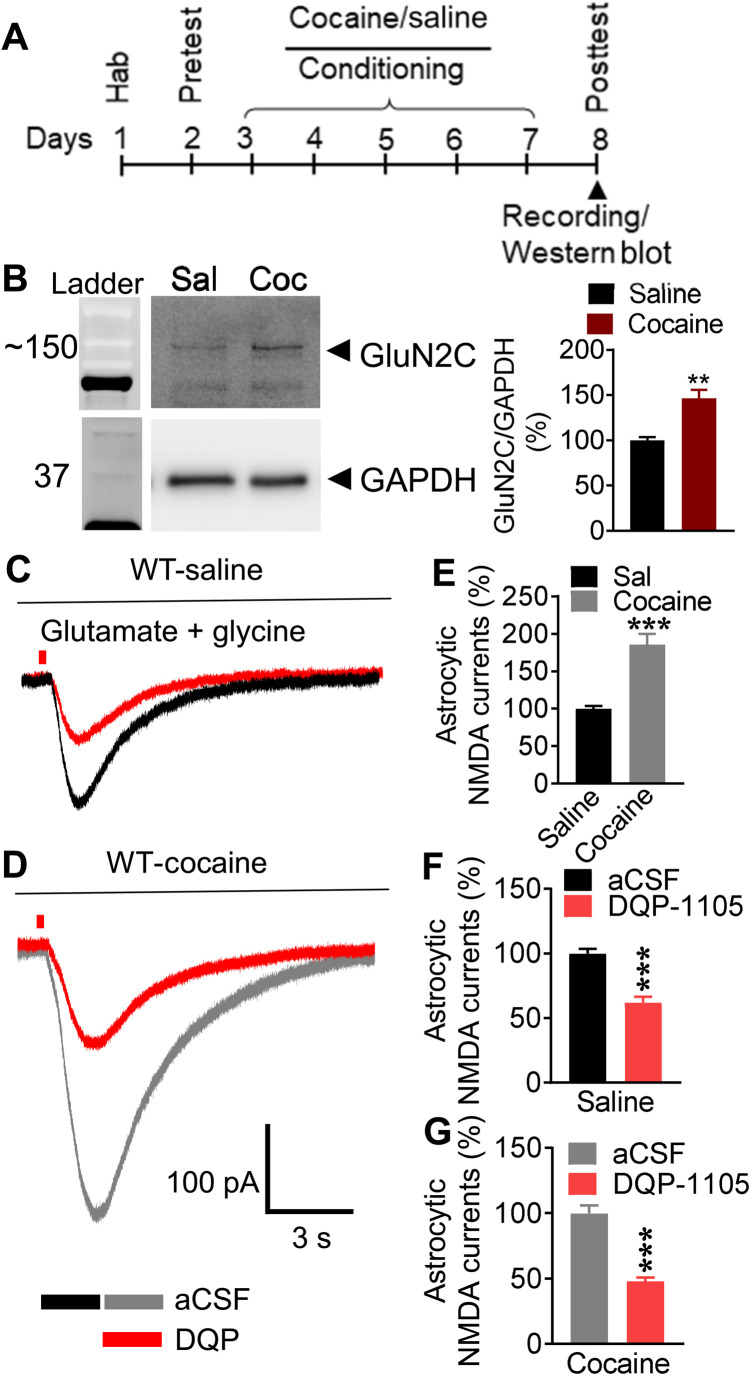
Repeated cocaine exposure increases GluN2C subunit expression and astrocytic NMDA receptor currents in the NAc core. (**A**) Experimental approach used for cocaine conditioning. (**B**) Cocaine conditioning up-regulates GluN2C expression in the NAc. Western blotting analysis of synaptoneurosomal preparation from WT mice following saline or cocaine conditioning. Significant up-regulation of GluN2C was observed after cocaine conditioning (**P = 0.0049, unpaired *t* test; *n* = 5). Whole-cell voltage-clamp recordings from NAc core astrocytes from saline-conditioned (**C**) and cocaine-conditioned (**D**) mice. Cocaine conditioning significantly increased the amplitude of NMDA receptor currents in astrocytes as compared to saline treatment (***P = 0.0003, unpaired *t* test; *n* = 9 per treatment) (**E**). GluN2C/2D antagonist DQP-1105 significantly reduced astrocytic NMDA receptor currents in saline-treated (***P = 0.0004, paired *t* test; *n* = 9) (**F**) and cocaine-treated (***P = 0.0007, paired *t* test; *n* = 9) (**G**) mice.

Knowing the selective and robust role of the astrocytic NMDA receptors in the extinction phase of cocaine CPP, we examined whether repeated cocaine exposure leads to changes in the expression of specific NMDA receptor subunits, enabling a greater role in extinction. Western blot analysis of NAc protein preparation after cocaine conditioning (or saline controls) revealed a significant up-regulation of GluN2C subunit upon repeated cocaine exposure (*P* = 0.0049, unpaired *t* test; Fig. 4, A and B). No significant change was noted in the expression of GluN1, GluN2A, GluN2B, or GluN2D subunits (*P* = 0.1497, *P* = 0.2978, *P* = 0.5823, and *P* = 0.4766, respectively, unpaired *t* test; fig. S4, A to D). To directly examine potential plasticity in astrocytic NMDA receptor currents upon repeated cocaine exposure and contribution of GluN2C subunit in this plasticity, we conducted electrophysiology analysis. NMDA receptor currents were recorded from astrocytes from brain slices obtained from cocaine (or saline control)–conditioned mice (Fig. 4, A and C to G). Notably, NMDA receptor–mediated currents in astrocytes were significantly higher in amplitude in cocaine-conditioned mice as compared to saline treatment (*P* = 0.0003, unpaired *t* test; Fig. 4, C to E). Bath application of GluN2C/2D-selective antagonist DQP-1105 significantly reduced the NMDA receptor currents in saline-treated (*P* = 0.0004, paired *t* test; Fig. 4, C and F) and cocaine-treated (*P* = 0.0007, paired *t* test; Fig. 4, D and G) mice. Notably, the percent inhibition by DQP-1105 was significantly higher in cocaine-treated mice compared to saline-treated mice (% response remaining after DQP-1105: saline; 61.96 ± 4.391 versus cocaine; 47.84 ± 2.945, *P* = 0.0183, unpaired *t* test). Thus, our results demonstrate plasticity of astrocytic GluN2C subunit–containing NMDA receptors upon repeated cocaine exposure, which may have implications for behavior.

### GluN2C subunit–containing receptors in the NAc are important for retention of cocaine memory

**Fig. 5.**
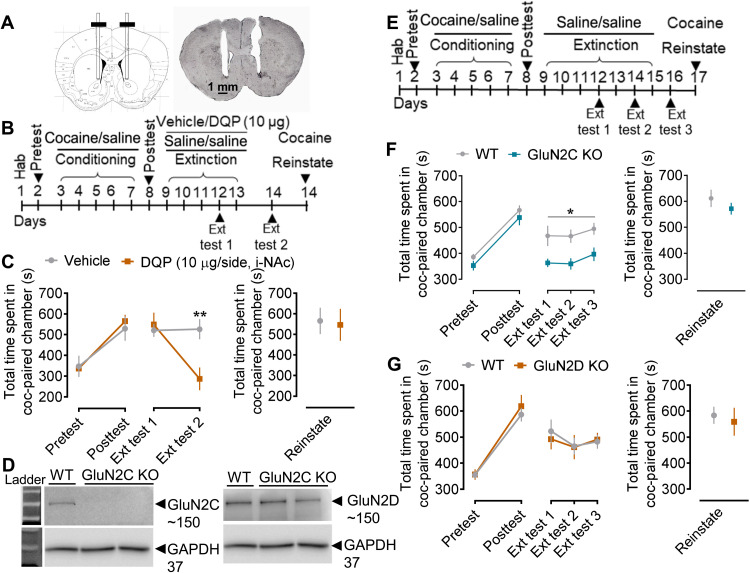
GluN2C subunit of the NMDA receptor is crucial for the maintenance of cocaine memory. (**A**) WT animals stereotaxically cannulated into the NAc core were tested for cocaine CPP. (**B**) Experimental design used for cocaine conditioning, extinction, and reinstatement experiments. (**C**) The intra-NAc (i-NAc) administration of DQP-1105 (10 μg per side, i-NAc) after each extinction session led to significant reduction in time spent in the cocaine-paired chamber during extinction (**P = 0.0068). Two-way ANOVA showed significant effect of DQP-1105 treatment on cocaine extinction (*F*_1,8_ = 5.36, *P* = 0.0493, two-way ANOVA; *n* = 5 each group). No differences in the two groups were observed during reinstatement (*P* = 0.3701, unpaired *t* test). (**D**) Immunoblot from NAc synaptoneurosomal preparation of WT and GluN2C KO mice, confirming loss of GluN2C subunit. (**E**) Experimental approach used for cocaine conditioning, extinction, and reinstatement experiments. (**F**) No significant difference in WT and GluN2C KO following cocaine conditioning (*P* = 0.8690, two-way ANOVA). GluN2C KO mice exhibit enhanced extinction with lower time spent in the cocaine-paired chamber compared to WT (*P = 0.0341, *P = 0.0292, and *P = 0.050 at extinction tests 1, 2, and 3, respectively). Two-way ANOVA showed significant effect of genotype on extinction (*F*_1,22_ = 9.544, *P* = 0.0054; *n* = 14 WT and 10 GluN2C KO). No differences in reinstatement were observed between the two groups (*P* = 0.9906, unpaired *t* test). (**G**) Effect of ablation of GluN2D subunit on cocaine place preference behavior. No significant differences were observed between WT and GluN2D KO during cocaine conditioning (*P* = 0.8884), extinction (*P* > 0.9999 each, two-way ANOVA), and reinstatement (*P* = 0.7037, unpaired *t* test). Two-way ANOVA showed no significant effect of ablation of GluN2D on cocaine extinction behavior (*F*_1,15_ = 0.0361, *P* = 0.8519; *n* = 8 WT and 9 GluN2D KO).

Given the selective and robust increase in GluN2C expression following cocaine conditioning (Fig. 4), we next tested the effect of pharmacological blockade of GluN2C/2D-containing receptors in the NAc core on cocaine CPP (Fig. 5, A to C). We stereotaxically cannulated WT mice bilaterally into the NAc core (Fig. 5A), and following 10 days of recovery period, these animals were tested for cocaine CPP behavior (Fig. 5B). Intra-NAc core administration of GluN2C/2D antagonist DQP-1105 (10 μg per side) after each extinction session significantly reduced preference for the cocaine side during second post-extinction test (*P* = 0.0068, two-way ANOVA). These results demonstrate a critical role of GluN2C/2D-containing receptors in the retention of cocaine preference memory. No difference was observed between the DQP-1105 and vehicle-treated groups in the reinstatement phase (*P* = 0.3701, unpaired *t* test).

To dissect the contributions of GluN2C versus GluN2D subunits in cocaine CPP, we used KO models. The lack of GluN2C subunit expression in the synaptoneurosome preparations in the GluN2C KO model was confirmed using immunoblotting (Fig. 5D). No significant difference in the time spent in the cocaine-paired chamber was observed after conditioning in GluN2C KO mice compared to WT (*P* = 0.8690; Fig. 5, E and F). Reduced time spent in the cocaine-paired chamber was observed in both WT and GluN2C KO mice after first extinction test (WT: *P* = 0.0015 and GluN2C KO: *P* < 0.0001 versus their respective conditioning group, two-way ANOVA; fig. S5, A and B), showing successful extinction. Notably, during extinction training, GluN2C KO mice showed a more robust extinction compared to the WT group (*P* = 0.0341, *P* = 0.0292, and *P* = 0.050 at extinction tests 1, 2, and 3, respectively, two-way ANOVA; Fig. 5F). Upon reinstatement with a single dose of cocaine, time spent in the cocaine-paired chamber reached levels similar to before extinction training. No difference in reinstatement was observed between the two groups (*P* = 0.9906, unpaired *t* test). Furthermore, to assess the sex-specific effects, we performed sex-split analysis in these mice. The sex-split analyses showed that sex did not produce a main effect; both GluN2C KO male and female mice showed significant facilitation of cocaine extinction (GluN2C KO-M: *P* = 0.0068, GluN2C KO-F: *P* = 0.0115; fig. S5F), suggesting that the sex differences do not underlie the observed role of NMDA receptors in astrocytes in regulating cocaine preference. Together, these data demonstrate that the GluN2C subunit contributes to retention of cocaine preference memory, and loss of GluN2C subunit accelerates extinction of cocaine preference.

Next, we examined the role of the GluN2D subunit in cocaine CPP using GluN2D KO mice (Fig. 5, E and G). WT and GluN2D KO mice showed similar levels of time spent in the cocaine-paired chamber in the posttest (*P* = 0.8884, two-way ANOVA) and during extinction (*P* > 0.9999 each, two-way ANOVA) and reinstatement (*P* = 0.7037, unpaired *t* test; Fig. 5G). Both groups showed reduced time spent in the cocaine-paired chamber following extinction training (average of all extinction posttests; fig. S5C), showing manifestation of extinction. Cocaine-induced locomotor sensitization was reduced in the GluN2D KO but not GluN2C KO mice as compared to WT control (*P* = 0.0117, two-way ANOVA; fig. S5, D and E). Together, our results suggest that GluN2D and GluN2C may regulate different aspects of cocaine behaviors.

### Astrocytic NMDA receptors regulate structural neuroadaptations in dendritic spines in the NAc

**Fig. 6.**
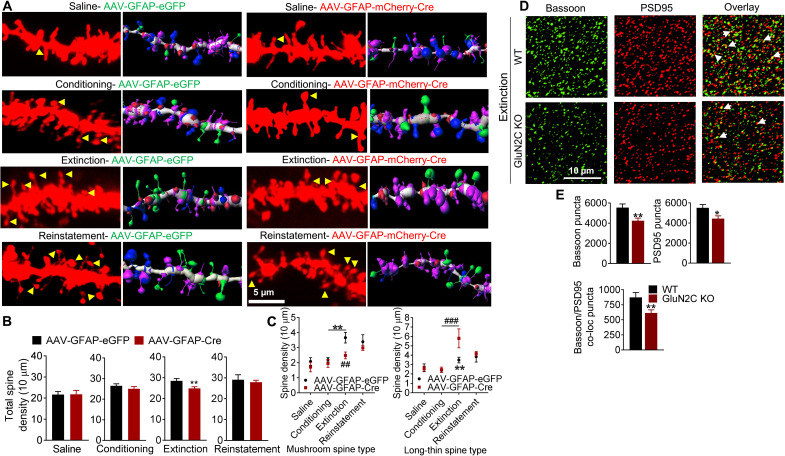
Cocaine-induced neuroadaptations in dendritic spines of NAc core medium spiny neurons require astrocytic NMDA receptors. (**A**) Representative confocal images of diolistically labeled and 3D reconstructed dendrites from AAV-control– and AAV-cre–injected GluN1^flox/flox^ mice following saline, cocaine conditioning, extinction, and reinstatement. Arrowheads indicate spines with head and neck. (**B**) Significantly lower total dendritic spine density in AAV-cre versus AAV-control animals in extinction (**P = 0.0089, unpaired *t* test; *n* = 5 mice per group). No changes were observed in saline (*P* = 0.9370), cocaine conditioning (*P* = 0.3417), and reinstatement groups (*P* > 0.05, unpaired *t* test). (**C**) Increase in mushroom-type spines after extinction in AAV-control (**P = 0.0032, two-way ANOVA with Bonferroni’s test) but not AAV-cre mice (*P* = 0.9697, two-way ANOVA). Significantly higher mushroom spines in AAV-control versus AAV-cre group after extinction (^##^*P* = 0.0030, two-way ANOVA). Two-way ANOVA revealed significant effect in AAV-control and AAV-cre groups (*F*_3,148_ = 9.448, *P* < 0.0001; *n* = 5 per group). Significant increase in long-thin spine type during extinction compared to conditioning in AAV-cre (^###^*P* = 0.0001) but not in AAV-control group (*P* > 0.9999, two-way ANOVA). Significant increase in long-thin spine following extinction in AAV-cre versus AAV-control groups (**P = 0.0059, two-way ANOVA). Two-way ANOVA revealed significant effect between AAV-control and AAV-cre groups (*F*_3,165_ = 6.393, *P* < 0.0004; *n* = 5 per group). (**D**) Confocal images from the NAc core of WT and GluN2C KO mice following extinction training immunostained for bassoon and PSD95. Arrowheads show colocalized puncta. (**E**) Quantification of immunostaining. Significantly lower puncta for bassoon (**P = 0.0046, unpaired *t* test), PSD95 (*P = 0.0141), and colocalized bassoon/PSD95 (**P = 0.0074) in GluN2C KO extinction compared to WT extinction group. Bar graphs represent mean ± SEM.

Cocaine has been found to induce neuroadaptations in NAc MSNs including the generation of silent synapses (*23*) and an increase in synaptic strength (*11*, *12*), which may underlie the formation of reinforcing memory. Cocaine exposure followed by withdrawal increases the diameter of spines, suggesting spine maturation (*24*, *25*). Because astrocytic processes ensheathe the dendritic spines, astrocytic NMDA receptors may affect spine plasticity, possibly accounting for its behavioral regulation of cocaine preference and extinction (*6*, *26*). To answer this possibility, we measured spine dynamics in GluN1^flox/flox^ mice stereotaxically injected with AAV-control or AAV-cre into the NAc core. Mice underwent saline or cocaine CPP training and were euthanized following conditioning posttest (for both saline and cocaine), extinction posttest (cocaine alone), or reinstatement posttest (cocaine alone), and the brains were processed for diolistic labeling, confocal imaging, and three-dimensional (3D) reconstruction to evaluate dendritic spine density and morphology (Fig. 6A). Saline-alone AAV-control and AAV-cre groups did not show any differences in spine density (*P* = 0.9370, unpaired *t* test; Fig. 6B) or type of spines (*P* > 0.05, two-way ANOVA; Fig. 6C). Thus, astrocytic NMDA receptor ablation alone does not produce any basal effect on dendritic spines in the NAc MSNs. Spine density appeared to increase after cocaine conditioning and extinction compared to saline control in AAV-control mice. No significant changes in spine density in AAV-cre versus AAV-control groups were observed following cocaine conditioning (*P* = 0.3417, unpaired *t* test; Fig. 6B). A lower total spine density was observed upon extinction in the AAV-cre group compared to AAV-control (*P* = 0.0089, unpaired *t* test; Fig. 6B). Extinction training led to an increase in mushroom spines in AAV-control mice compared to conditioning (*P* = 0.0032, two-way ANOVA; Fig. 6C), suggesting spine maturation. However, this was not the case in mice with astrocytic NMDA receptor ablation (AAV-cre) (*P* = 0.9697, two-way ANOVA; Fig. 6C). A significant difference in mushroom spines was observed between AAV-control and AAV-cre post-extinction groups (*P* = 0.0030, two-way ANOVA). Furthermore, extinction training significantly increased long-thin spines in AAV-cre mice compared to conditioning (*P* = 0.0001, two-way ANOVA; Fig. 6C). In addition, long-thin spines were significantly higher in AAV-cre during extinction phase compared to AAV-control (*P* = 0.0059, two-way ANOVA; Fig. 6C). No difference was observed in spine density or type of spines in the two groups during reinstatement (*P* > 0.05, two-way ANOVA). These results are in parallel with our behavioral results, which show a selective role of astrocytic NMDA receptors in the maintenance of cocaine memory during extinction, with no significant differences in conditioning or reinstatement. Furthermore, these results suggest that spine maturation may serve as a molecular correlate for the maintenance of cocaine memory, which requires astrocytic NMDA receptor function. This finding is also consistent with recent findings with cocaine and heroin self-administration and extinction, demonstrating spine maturation (*27*). We also assessed mature synapse numbers using PSD95-bassoon colocalization in WT and GluN2C KO mice (Fig. 6D). A significantly lower number of colocalized synaptic puncta were noted in GluN2C KO compared to WT in the extinction group (*P* = 0.0074, unpaired *t* test; Fig. 6E) but not in conditioning (fig. S6, A and B). These results are consistent with the observations in spine morphology analysis, demonstrating higher mature spine count in WT extinction group but not following ablation of astrocytic NMDA receptors.

### Astrocytic NMDA receptors regulate cocaine-induced synaptic neuroadaptations

**Fig. 7.**
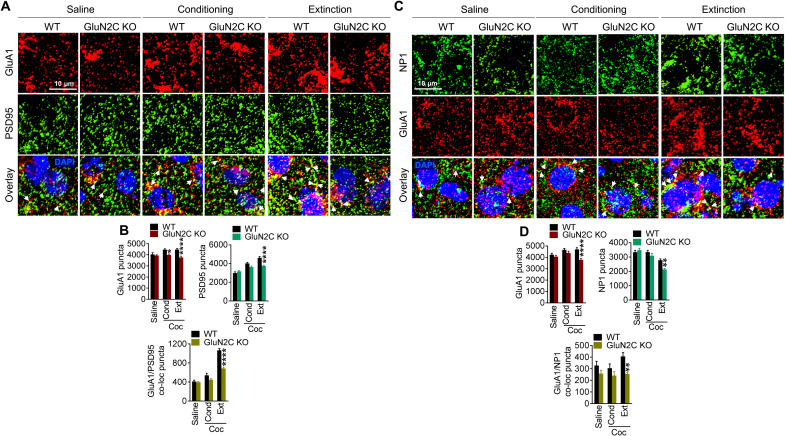
GluN2C subunit regulates cocaine-induced synaptic strengthening via synaptic clustering of GluA1 AMPA receptors. (**A**) Representative confocal images from the NAc core immunostained for GluA1 and PSD95 after saline, cocaine conditioning, or extinction treatment in WT and GluN2C KO mice. DAPI, 4′,6-diamidino-2-phenylindole. (**B**) Quantification of immunostaining. Significant difference in WT versus GluN2C KO mice in GluA1 puncta (conditioning: *P = 0.0244, extinction: ****P < 0.0001; *F*_5,195_ = 7.835, *P* < 0.0001, one-way ANOVA with Bonferroni’s test), PSD95 puncta (extinction: ****P < 0.0001; *F*_5,194_ = 25.85, *P* < 0.0001, one-way ANOVA with Bonferroni’s test), and GluA1/PSD95 colocalized puncta (extinction: ****P < 0.0001; *F*_5,190_ = 76.89, *P* < 0.0001, one-way ANOVA with Bonferroni’s test). (**C**) Confocal images from the NAc core of WT and GluN2C KO mice immunostained for NP1 and GluA1 after saline, cocaine conditioning, or extinction. (**D**) Quantification of immunostaining. Significant difference in post-extinction WT versus GluN2C KO mice in GluA1 puncta (****P < 0.0001; *F*_5,199_ = 6.821, *P* < 0.0001, one-way ANOVA with Bonferroni’s test), NP1 puncta (**P = 0.0019; *F*_5,174_ = 18.72, *P* < 0.0001, one-way ANOVA with Bonferroni’s test), and GluA1/NP1 colocalized puncta (**P = 0.0062; *F*_5,178_ = 4.096, *P* = 0.0015, one-way ANOVA with Bonferroni’s test). Bar graphs represent mean ± SEM, each data point representing 32 to 34 images from *n* = 5 WT and *n* = 5 GluN2C KO mice.

Cocaine exposure is known to induce synaptic plasticity primarily exemplified by an increase in AMPA receptor expression (*28*). We examined changes in the expression of AMPA receptor subunit GluA1 in the NAc core using immunohistochemistry upon conditioning and extinction in WT and GluN2C KO mice (Fig. 7, A and B). GluA1 puncta were significantly lower in GluN2C KO mice compared to WT mice during conditioning (*P* = 0.0244) and extinction (*P* < 0.0001, one-way ANOVA; Fig. 7B), as compared to WT mice. Synaptic stabilization of GluA1 AMPA receptors at synapses is an important mechanism in synaptic plasticity, which is supported by PSD95 (*29*). We found that, in WT mice, cocaine conditioning and extinction significantly increased PSD95 puncta (*P* < 0.0001 each, one-way ANOVA) and GluA1/PSD95 colocalized puncta (conditioning: *P* = 0.0437; extinction: *P* < 0.0001, one-way ANOVA; Fig. 7B), as compared to saline treatment. The increase was particularly robust in extinction, suggesting plasticity changes that may contribute to retention of cocaine preference memory. Compared to WT mice, GluN2C KO mice that underwent extinction training had lower PSD95 puncta (*P* < 0.0001) and GluA1/PSD95 colocalized puncta (*P* < 0.0001, one-way ANOVA; Fig. 7B). We also assessed the changes in neuronal pentraxin 1 (NP1) (Fig. 7, C and D), which is responsible for recruitment of AMPA receptors to the synapses (*30*). Consistent with the changes noted with GluA1 expression, NP1 was found to be down-regulated in GluN2C KO during the extinction phase (*P* < 0.0001, one-way ANOVA). Significantly lower NP1 puncta (*P* = 0.0019, one-way ANOVA) and GluA1-NP1 (*P* = 0.0062; Fig. 7D) colocalized puncta were observed in GluN2C KO compared to WT during extinction.

**Fig. 8.**
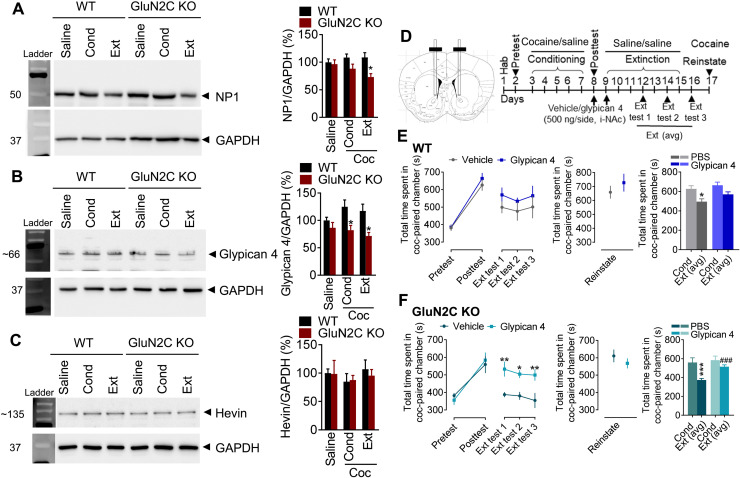
Astrocytic GluN2C-containing NMDA receptors regulate glypican 4 and NP1 expression. (**A** to **C**) Western blot analysis of NAc synaptoneurosome preparation from WT and GluN2C KO mice. (A) Significant down-regulation of NP1 in GluN2C KO mice compared to WT following cocaine extinction (*P = 0.0254; *n* = 5 each group per genotype) (*F*_5,38_ = 3.365, *P* = 0.0130; *n* = 7 to 8 each group per genotype; one-way ANOVA). (B) Significant decrease in glypican 4 expression in GluN2C KO compared to WT following conditioning and extinction (*P = 0.0485 and *P = 0.0372, respectively) (*F*_5,34_ = 4.572, *P* = 0.0027; *n* = 6 to 8 each group per genotype; one-way ANOVA). (C) No significant change in hevin in GluN2C KO mice following conditioning or extinction (*P* > 0.05; *n* = 5 to 7 each group per genotype). (**D**) Experimental strategy to test the effect of glypican 4 (500 ng per side, i-NAc). (**E**) Glypican 4 treatment in WT mice produced no significant change in time spent in the cocaine-paired chamber as compared to vehicle treatment (*P* > 0.05; *n* = 7 each; two-way ANOVA). Bar graphs showing significantly reduced time spent in the cocaine-paired chamber during extinction in vehicle-treated (*P = 0.0499) but not glypican 4–treated mice (*P* = 0.4314, one-way ANOVA). (**F**) Glypican 4 treatment in GluN2C KO mice significantly increased time spent in the cocaine-paired chamber during extinction test as compared to vehicle-treated mice (**P = 0.0085, *P = 0.0271, and **P = 0.0086 at extinction tests 1, 2, and 3, respectively). Two-way ANOVA showed significant effect of glypican 4 treatment on extinction of cocaine CPP in GluN2C KO mice (*F*_1,9_ = 23.22, *P* = 0.0009; *n* = 6 each treatment). Bar graphs showing significantly reduced time spent in the cocaine-paired chamber in the vehicle group (***P = 0.0002, one-way ANOVA), which was significantly increased following glypican 4 treatment (^###^*P* = 0.0002, one-way ANOVA).

We next examined the potential effect of astrocytic NMDA receptors on synaptogenic molecules, which may underlie their effect on cocaine-induced plasticity. NP1, glypican 4, and hevin expression were evaluated in WT that underwent CPP (Fig. 8, A to C). Similar to puncta analysis, a significantly lower NP1 expression was observed post-extinction in GluN2C KO compared to WT (*P* = 0.0254, one-way ANOVA; Fig. 8A). Expression of astrocytic released glypican 4 modestly increased after cocaine conditioning in WT mice. In contrast, GluN2C KO had significantly lower post-conditioning and post-extinction levels of glypican 4 expression compared to WT (*P* = 0.0485 and *P* = 0.0372, respectively, one-way ANOVA; Fig. 8B). No significant changes in hevin expression were observed in either of the groups (*P* > 0.05 each; Fig. 8C). Together, these results demonstrate that the accelerated extinction in GluN2C KO mice is because of impaired synaptic maturation and down-regulation of synaptic AMPA receptors potentially mediated by reduced recruitment of synaptogenic factors NP1 and glypican 4.

### Intra-NAc administration of recombinant glypican 4 restored the deficit in retention of cocaine memory in GluN2C KO mice

Knowing the specific changes in glypican 4 expression during post-extinction phase (Fig. 8B), we next asked whether supplementing glypican 4 exogenously would enhance retention of cocaine preference memory. To answer this possibility, we performed stereotaxic cannulations into the NAc core of WT and GluN2C KO mice and performed cocaine CPP (Fig. 8D). Recombinant glypican 4 (500 ng per side, intra-NAc) was administered into the NAc of WT and GluN2C KO mice after conditioning posttest and after the first extinction session. In the GluN2C KO mice, vehicle-treated mice exhibited significant extinction (average of all extinction posttests, *P* < 0.0002, one-way ANOVA; Fig. 8F). In contrast, glypican 4–treated GluN2C KO mice spent significantly more time in the cocaine-paired chamber during extinction test as compared to vehicle-treated mice, suggesting rescue of impairment in retention of cocaine preference memories (*P* = 0.0085, *P* = 0.0271, and *P* = 0.0086 at extinction tests 1, 2, and 3, respectively; Fig. 8F). In the WT mice, no significant differences in vehicle versus glypican 4 groups were observed (*P* > 0.05, one-way ANOVA; Fig. 8E).

## DISCUSSION

Converging findings have demonstrated the existence of NMDA receptors in nonneuronal cells, but their function in plasticity and behavior is largely unknown. Here, we found that astrocytic NMDA receptors in the NAc play a critical role in the retention of cocaine memory by regulating dendritic spine and synaptic neuroadaptations potentially mediated by release of astrocytic synaptogenic factors. This report demonstrates that NMDA receptors in astrocytes are active participants in drug-induced plasticity, and given the widespread expression of astrocytic NMDA receptors, similar mechanisms may subserve other forms of plasticity and behavioral control.

### Astrocytes in NAc express GluN2C subunit–containing NMDA receptors with GluN1/2A/2C composition

Over the past four decades, the existence of NMDA receptors in astrocytes has remained controversial, and their functional roles have been largely overlooked. Recently, using a eGFP reporter mouse for the *GRIN2C* gene, we have demonstrated the expression of GluN2C subunits in astrocytes but not in neurons, microglia, or gamma-aminobutyric acid (GABA)ergic neurons in several corticolimbic regions including the striatum, cortex, and basolateral amygdala (*16*). In the present study using this reporter model, we provided evidence for the presence of GluN2C subunits in NAc astrocytes. We further established the expression of GluN2C subunit–containing receptors in whole-cell recordings. Application of glutamate + glycine or electrical stimulation (isolated by using blockers to inhibit currents from other receptors and transporters) produced NMDA receptor currents in NAc astrocytes, which were reduced by ~50% by bath application of GluN2C/2D antagonist DQP-1105. In GluN2C KO mice, the astrocytic NMDA receptor currents were significantly smaller in amplitude and were insensitive to DQP-1105. NMDA receptor currents from MSNs were insensitive to DQP-1105, suggesting that they lacked GluN2C/2D subunits. We also identified a contribution of GluN2A but not GluN2B subunit to the NMDA receptor currents in astrocytes. Because most of the NMDA receptor currents in astrocytes were blocked by a combination of DQP-1105 and TCN201, we predict the composition of astrocytic NMDA receptors to be GluN1/2A/2C. This composition has similarity to other regions, where GluN2C is expressed in particular cerebellar granule cells (*31*). Consistent with previous studies that have found that astrocytic NMDA receptors are only weakly blocked by Mg^2+^ (*14*, *15*), our recording conditions also suggest weak Mg^2+^ sensitivity of astrocytic NMDA receptor currents. Overall, using electrophysiology, pharmacology, and genetic tools, our study unequivocally demonstrates the expression of GluN2C subunit–containing NMDA receptors in astrocytes.

### Cocaine experience induces plasticity of astrocytic NMDA receptors in the NAc

Cocaine exposure has been shown to induce several neuroadaptations in NAc including an increase in glutamate receptor expression (*11*, *12*), which may underlie the formation of new drug-associated memories. We found that the amplitude of astrocytic NMDA receptor currents and the contribution of GluN2C subunit to these currents (assessed by DQP-1105 inhibition) increased significantly in cocaine-conditioned mice. In addition, we observed an increase in the expression of GluN2C subunit in immunoblotting after cocaine conditioning. Thus, these results demonstrate plasticity of astrocytic GluN2C subunit–containing NMDA receptors in NAc following repeated cocaine exposure without the requirement for abstinence, which may have implications for the maintenance of cocaine preference memories. Note that one previous report has found that cocaine exposure followed by abstinence increases GluN2C/2D NMDA receptor currents at the thalamostriatal synapses in MSNs (*32*). Although the authors suggested the contribution of the GluN2C subunit in this phenomenon, because of the lack of specificity of pharmacological tools and the lack of use of genetic models, the contribution of GluN2C versus GluN2D could not be distinguished. Our previous and current results demonstrate that GluN2C subunits are expressed in astrocytes in the NAc and are unlikely to directly mediate enhanced thalamostriatal NMDA receptor function and, instead, GluN2D may underlie this phenomenon.

### Selective role of astrocytic NMDA receptors in extinction of cocaine preference

Astrocytes are effective modulators of neuronal activity, and their role in synapse formation, maintenance, and elimination has been demonstrated (*8*). Although cocaine-associated reward and addiction are primarily linked to neuronal circuit activities, increasing body of evidence suggests a dynamic role of astrocytes in cocaine-induced maladaptive changes at glutamatergic synapses in the NAc (*7*, *33*, *34*). We found that ablation of the obligatory GluN1 subunit from astrocytes facilitated extinction of cocaine preference memory without affecting conditioning or reinstatement (Figs. 2 and 3). Notably, cocaine-induced reinstatement led to a complete recovery of preference to preextinction levels, suggesting that the original memory was still intact. We found that astrocytic NMDA receptors in the NAc core, but not in PFC, facilitate extinction of cocaine CPP, suggesting the region-specific effect of astrocytic NMDA receptors. It is possible that the lack of behavioral effects in PFC might be due to the regional heterogeneity of astrocytes such as different morphological, proteomic, transcriptomic, and functional profiles of the astrocytes in these regions (*35*). A change in expression of GFAP, total surface area and astrocytic volume, and altered synaptic contacts of astrocytic processes with presynaptic nerve terminal marker synapsin-1 was observed during extinction of cocaine self-administration in the NAc astrocytes (*6*) but not in PFC and the basolateral amygdala (*36*), indicating the selective response of NAc astrocytes in extinction learning. Furthermore, dopamine concentration in NAc versus PFC after cocaine injection may result in adaptations in glutamatergic and GABAergic signaling that may well contribute to the region-specific effects observed.

These effects of astrocytic NMDA receptors are in contrast to the roles designated to neuronal NMDA receptors in a drug-induced behavior. Several pharmacological and genetic models have found that inhibition of NMDA receptors in the NAc prevents or reduces cocaine CPP (*37*) and cocaine self-administration (*38*) and attenuates cue-induced reinstatement of cocaine seeking (*39*). Ablation of NMDA receptors from D1 MSNs also attenuates both drug-induced CPP and locomotor sensitization (*40*). Furthermore, adult ablation of GluN1 subunits from D1 MSNs completely eliminates cocaine conditioning (*41*). Thus, the selective effect of GluN1 ablation from astrocytes on extinction is unique compared to neuronal roles. The initial formation of cocaine preference memory appears to be independent of astrocytic NMDA receptors. Instead, cocaine experience appears to prime the astrocytes to participate in strengthening of cocaine memory by increasing GluN2C expression in astrocytes. It is likely that cocaine exposure initiates other changes in astrocytes and ambient neurotransmitter levels that, together with astrocytic NMDA receptors, are important for strengthening of cocaine memory.

### GluN2C subunit of NMDA receptors regulate the extinction of cocaine preference

GluN2C- and GluN2D-containing receptors share certain common biophysical and pharmacological properties (*42*) but show different patterns of regional and cell type–specific expression especially in the striatum (*16*). We found that intra-NAc administration of GluN2C/2D inhibitor DQP-1105 facilitated extinction of cocaine memory. Our complementary electrophysiology experiments suggest that intra-NAc DQP-1105 preferentially inhibits astrocytic GluN2C receptors. This is supported by the findings that DQP-1105 inhibition of NMDA receptor currents in astrocytes was GluN2C dependent (inhibition was lost in GluN2C KO) and DQP-1105 did not affect NMDA receptor currents in MSNs. We further found that GluN2C KO, but not GluN2D KO, mice showed facilitation of extinction of cocaine CPP. Together, the molecular and behavioral analysis demonstrates that repeated cocaine exposure up-regulates the GluN2C subunit, which supports the retention of cocaine memory during the extinction phase. We also found that GluN2D KO mice showed reduced cocaine-induced locomotor sensitization. Locomotor sensitization is dependent on the generation of GluN2B-containing silent synapses in the NAc (*43*), and generally, GluN2D expresses as triheteromer combinations with GluN2B (*44*). Thus, GluN2C and GluN2D subunits may play unique cell type–specific roles depending on the nature of cocaine exposure and may be responsible for different behavioral outcomes.

### Astrocytic NMDA receptors are obligatory for cocaine experience–induced dendritic spine maturation and synaptic strengthening in the NAc

Cocaine exposure induces neuroadaptations in the NAc including the generation of silent synapses and synaptic strengthening (*11*, *12*). Cocaine exposure followed by withdrawal increases the diameter of spines, suggesting spine maturation (*24*, *25*). Consistent with previous observations, we found that, in control animals (AAV-eGFP), cocaine conditioning followed by extinction training increased mushroom-type spines, which represent mature spines. However, such adaptation was absent in the mice with conditional astrocytic NMDA receptor ablation (AAV-cre). In contrast, significantly higher numbers of long-thin immature spines and lower total spine density were noted in AAV-cre mice in extinction. Changes in dendritic spine types mirrored the changes in behavior during CPP. Specifically, spine morphology and density changes were noted in the AAV-cre group compared to control at post-extinction time point but not after conditioning or reinstatement. The increase in mushroom-type spines after extinction in WT mice also correlated with an increase in synaptic GluA1 subunits, which was not the case in GluN2C KO mice. Specifically, an increment in GluA1-PSD95 puncta was observed post-extinction in WT animals, consistent with the stabilization of synapses. The degree of increase in GluA1-PSD95 puncta post-extinction was significantly lower in GluN2C KO mice. It is well established that repeated exposure to cocaine followed by withdrawal/abstinence increases the AMPA receptor expression or currents in the NAc MSNs. However, most of these studies have found a requirement for a long withdrawal (3 weeks or longer). Nonetheless, some studies have observed this phenomenon after a shorter withdrawal. For example, an increase in surface AMPA expression or AMPA/NMDA ratio was observed after 7 days or 10 to 14 days of withdrawal (*12*, *45*, *46*). Note that we did not observe a robust change in GluA1 puncta alone (noncolocalized) in WT mice after conditioning or extinction, but an increase in GluA1-PSD95 colocalized puncta after 7 days of extinction training is very robust. It is possible that this additional difference in the methodology may provide differential sensitivity in detecting change in GluA1 distribution. It has been found that GluA1-containing AMPA receptors are preferentially incorporated into mushroom-type dendritic spines, thereby stabilizing synapses following learning (*47*). Furthermore, studies have identified a role of NP1 in recruitment of AMPA receptors to the synapse (*30*). We found reduced NP1 recruitment in GluN2C KO mice. Together, these results demonstrate that the maturation of spines and NP1-mediated recruitment of AMPA receptors following cocaine experience require the function of astrocytic GluN2C NMDA receptors.

How may astrocytic NMDA receptors participate in synaptic strengthening? It has been established that astrocyte-secreted factors such as hevin, chordin-like 1, and glypican 4 promote synapse formation and maturation (*8*). Specifically, glypican 4 promotes the formation of excitatory synapses via GluA1 AMPA receptors (*48*). We examined changes in hevin and glypican 4 in CPP groups. No change in hevin expression was observed. However, glypican 4 was significantly lower post-extinction in GluN2C KO mice. Exogenous administration of glypican 4 in the NAc in GluN2C KO mice restored the deficit in retention of cocaine preference memory. These studies provide a clear causal link between GluN2C subunit–containing astrocytic NMDA receptor and glypican 4–induced synaptic strengthening in cocaine preference memory.

Although the expression of NMDA receptors in astrocytes is now supported by multiple lines of data, the precise localization at an ultrastructural level remains unknown. It can be envisioned that astrocytic NMDA receptors are expressed at astrocytic processes that ensheathe synapses and are activated in response to synaptic glutamate release. Repeated cocaine exposure, which causes an increase in astrocytic NMDA receptors, may cause stronger activation of these receptors and induce morphological changes in astrocytes such as retraction from synaptic elements, which has been observed upon cocaine and heroin extinction (*7*, *33*, *34*). NMDA receptor activation in neurons is associated with actin cytoskeleton remodeling (*49*), and similar processes may be engaged in astrocytes. Excess activation of astrocytic NMDA receptors upon drug exposure may also trigger signaling mechanisms underlying up-regulation or release of astrocyte-generated synaptogenic molecules, which may thereby mediate drug-induced plasticity. Our study provides initial clues by identifying glypican 4 as an astrocytic NMDA receptor–responsive factor that may mediate the maintenance of cocaine preference memory. In conclusion, we have identified a unique role of astrocytic GluN2C subunits and uncovered a previously unidentified molecular mechanism underlying cocaine extinction. GluN2C-containing astrocytic NMDA receptors are essential in regulating synaptogenic cues, which play a pivotal role in synapse maturation following cocaine exposure. Ablation of astrocytic NMDA receptors impairs these cues and prevents synaptic strengthening, thereby facilitating extinction of cocaine preference. Extinction of cocaine preference memories can help prevent craving and subsequent relapse. Thus, together with the recent drug discovery efforts focused on identifying GluN2C-selective potentiators and superagonists (*50*–*52*), these findings may have important therapeutic implications in preventing drug-induced craving and cocaine addiction.

## MATERIALS AND METHODS

### Animals

WT, Grin2C^tm1(EGFP/cre/ERT2)Wtsi^ (KO-first allele, GluN2C KO), and Grin2D^tm1a(EUCOMM)Wtsi^ (KO-first allele, GluN2D KO) mice (Wellcome Trust Sanger Institute), all on pure C57BL/6N background as described previously (*16*, *53*), were used for the present study. In addition, GluN1^flox/flox^ mice (congenic C57BL/6J; 005246, The Jackson Laboratory) and Aldh1L1cre/ERT2 (congenic C57BL/6N; 031008, The Jackson Laboratory) were used. The GluN1^flox/flox^ mice were crossed with Aldh1L1cre/ERT2 driver mice with tamoxifen-inducible cre recombinase expression directed at astrocytes. Mice from both sexes were used for experiments. Behavioral procedures were performed on age-matched 8- to 10-week-old mice. Animals were housed at constant temperature and 12-hour light/12-hour dark cycle with ad libitum access to food and water as described previously (*53*). All procedures were approved by the Creighton University Institutional Animal Care and Use Committee and conformed to the National Institutes of Health (NIH) *Guide for the Care and Use of Laboratory Animals*.

### Drugs

Cocaine HCl (Sigma-Aldrich, St. Louis, MO, USA) was dissolved in sterile saline and injected by intraperitoneal route (15 mg/kg). Tamoxifen (Sigma-Aldrich) was dissolved in corn oil (Sigma-Aldrich) at a concentration of 20 mg/ml by shaking overnight at 37°C and was administered by intraperitoneal route once a day for 5 days (75 mg/kg). Corn oil was used in the control group as vehicle. DQP-1105 (Tocris Bioscience, MN, USA) was dissolved in a mixture of dimethyl sulfoxide (DMSO) and polyethylene glycol (PEG) as a stock, and final dilutions (to 10 μg/0.3 μl) were made in PEG for injection into the NAc. Recombinant human glypican 4 (R&D Systems Inc., MN, USA) was dissolved in a sterile phosphate-buffered saline and injected into the NAc.

### Immunohistochemistry

Mice were deeply anesthetized with isoflurane and transcardially perfused with 4% paraformaldehyde (PFA) in 0.1 M phosphate buffer (PB). Brains were collected and processed for immunohistochemistry. Briefly, the brains were sectioned at 25 μM thickness through the NAc or PFC using a cryostat (Leica CM 1900, Buffalo Grove, IL). The sections were washed three times for 5 min each with 0.1 M PB and incubated in a blocking solution containing 10% normal goat serum or normal donkey serum in 0.25% Triton X-100 in 0.1 M PB (PBT) for 1 hour at room temperature. Following blocking, sections were incubated overnight at 4°C in the following primary antibodies: rabbit anti-GFAP (1:1000; G9269, Sigma-Aldrich, St. Louis, MO, USA, RRID: AB_477035), mouse anti-NeuN (1:200; MAB377, Millipore, MA, USA, RRID: AB_2298772), rabbit anti-Iba1 (1:1000; 019-19741, Wako Chemicals, USA, RRID: AB_839504), mouse anti-PSD95 (1:300; MA1-046, Invitrogen, USA, RRID: AB_2092361), mouse anti-bassoon (1:100; sc-58509, Santa Cruz Biotechnology, Dallas, TX, USA), rabbit anti-GluR1 (1:1000; 05-855R, Millipore, MA, USA, RRID: AB_1587070), chicken anti-GFP (1:1000; A10262, Thermo Fisher Scientific, Waltham, MA, USA, RRID: AB_2534023), and mouse anti-NP1 (1:100; sc-374199, Santa Cruz Biotechnology, Dallas, TX, USA) in solution containing 5% normal goat serum and/or donkey serum in PBT overnight at 4°C. The following day, sections were washed six times for 5 min each in 0.1 M PBT and incubated for 2 hours at room temperature in the dark with one or a combination of the following 488 and/or 594 fluorescently labeled secondary antibodies: goat anti-chicken conjugated to DyLight 488 (1:500; 072-03-24-06, KPL antibodies, IL, USA), goat anti-mouse conjugated to Alexa Fluor 594 (1:500; A11005, Thermo Fisher Scientific, RRID: AB_2534073), goat anti-rabbit conjugated to Alexa Fluor 594 (1:500; A11012, Thermo Fisher Scientific, RRID: AB_141359), donkey anti-mouse conjugated to Alexa Fluor 488 (1:500; A21202, Thermo Fisher Scientific, RRID: AB_141607), or goat anti-rabbit conjugated to Alexa Fluor 488 (1:500; A11008, Thermo Fisher Scientific, RRID: AB_143165). Sections were then washed three times for 5 min each in 0.1 M PBT, mounted on precleaned glass slides, and coverslipped with Fluoromount-G (0100-01, SouthernBiotech, Birmingham, AL). Confocal images were acquired using a Nikon Ti-E inverted microscope with a Yokagawa spinning disc. For puncta analysis, images of an equivalent region, 1024 pixels by 1024 pixels, were captured using a 60×, oil immersion objective at a 1× zoom. NAc core sections were scanned at 0.3-μm intervals along the *z* axis, and an optical section (7.2 μm thick) was taken from each tissue section. Colocalization and puncta number were analyzed by Volocity (PerkinElmer Inc., Coventry, United Kingdom). A total of 30 to 35 images from each CPP group (*n* = 5 mice per group per treatment) or from AldhCre-GluN1^flox^ vehicle and tamoxifen group (*n* = 5 per group) and naïve WT and GluN2C KO mice (*n* = 3 per genotype) were analyzed. Images were analyzed by a trained observer blind to genotypes.

### Electrophysiology

Whole-cell electrophysiology was performed as previously described (*50*, *54*). After isoflurane anesthesia, mice were decapitated, and brains were removed rapidly and placed in ice-cold aCSF of the following composition: 130 mM NaCl, 24 mM NaHCO_3_, 3.5 mM KCl, 1.25 mM NaH_2_PO_4_, 2.4 mM CaCl_2_, 2.5 mM MgCl_2_, and 10 mM glucose saturated with 95% O_2_/5% CO_2_. Sagittal sections (300 μm thick) were prepared using a vibrating microtome (Leica VT1200, Buffalo Grove, IL, USA). Whole-cell recordings were obtained in the NAc core from labeled astrocytes or neighboring neurons in voltage-clamp configurations with Axopatch 200B (Molecular Devices, Sunnyvale, CA, USA). Glass pipettes with a resistance of 3 to 5 megaohms were used. Signal was filtered at 2 kHz and digitized at 10 kHz using an Axon Digidata 1440A analog-to-digital board (Molecular Devices, CA). Whole-cell recordings with a pipette access resistance less than 20 megohms and that changed less than 20% during the duration of recording were included. For voltage-clamp recordings, glass pipettes were filled with an internal solution consisting of 126 mM cesium methanesulfonate, 8 mM NaCl, 10 mM Hepes, 8 mM Na_2_-phosphocreatine, 0.3 mM Na_2_GTP, 4 mM MgATP, 0.1 mM CaCl_2_, and 1 mM EGTA (pH 7.3). QX-314 (2.9 mM) was added to block voltage-gated sodium channels in recorded cells. Astrocytic NMDA receptor currents at −70 mV were evoked by picospitzer application (10 to 20 ms) of 1 mM glutamate + 100 μM glycine through a borosilicate capillary tube (1 to 2 megaohms) or stimulation of cortical inputs by a bipolar tungsten electrode (World Precision Instruments, FL, USA) in the presence of Tetrodotoxin (TTX, 30 μM), 6-cyano-7-nitroquinoxaline-2,3-dione (CNQX, 10 μM), DL-threo-beta-Benzyloxyaspartate (DL-TBOA, 30 μM), 2-amino-5,6,7,8-tetrahydro-4-(4-methoxyphenyl)-7-(1-naphthalenyl)-5-oxo-4H-1-benzopyran-3-carbonitrile (UCPH, 10 μM), pyridoxalphosphate-6-azophenyl-2’,4’-disulfonic acid (PPADS, 30 μM). Five stable baseline measurements were obtained followed by bath application of DL-AP5 (100 μM), DQP-1105 (20 μM), DQP-1105 + Ro25-6981 (1 μM), DQP-1105 + Ro25-6981 + TCN201 (10 μM), or DQP-1105 + Ro25-6981 + TCN201 + DL-AP5. For neurons, NMDA receptor currents were obtained by picospitzer application of 1 mM glutamate + 100 μM glycine through a borosilicate capillary tube (1 to 2 megaohms) or by stimulation of cortical inputs (~1.5 mA for 100 μs) and recordings were obtained at +40 mV in the presence of picrotoxin and CNQX.

### Tissue preparation and Western blotting

For sample preparation, mice were deeply anesthetized and decapitated, brains were isolated, and tissues were collected from the NAc region. Synaptoneurosome samples were prepared, and Western blotting was conducted as previously described (*53*–*55*). Briefly, samples were resolved on SDS–polyacrylamide gel electrophoresis gel and transferred onto nitrocellulose membrane. Membranes were blocked with 5% dry milk in tris-buffered saline/Tween 20 (TBST) at room temperature for 1 hour and incubated with an appropriate primary antibody overnight at 4°C. The specific antibodies used were as follows: rabbit anti–glypican 4 (1:300; Proteintech, 13048-1-AP, RRID: AB_10640157), goat anti-hevin (1:2000; R&D Systems, AF2836, RRID: AB_2195097), mouse anti-NP1 (1:1000; Santa Cruz Biotechnology, sc-374199), mouse anti-GluN2C (1:500; NeuroMab, 75-411, RRID: AB_2531892), rabbit anti-GluN1 (1:1000; Frontier Institute, AF720, RRID: AB_2571604), rabbit GluN2A (1:1000; Millipore, AB1555P, RRID: AB_310837), mouse GluN2B (1:1000; Millipore, 06-600, RRID: AB_310193), mouse GluN2D (1:1000; Millipore, MAB5578, RRID: AB_11214297), or mouse anti–glyceraldehyde phosphate dehydrogenase (GAPDH) (1:2000; Millipore, MAB374, RRID: AB_2107445). The blots were incubated in horseradish peroxidase–conjugated anti-rabbit (1:10,000; The Jackson Laboratory, 111-035-003, RRID: AB_2313567), anti-mouse (1:10,000; The Jackson Laboratory, 115-035-003, RRID: AB_10015289), or anti-goat secondary antibody (1:3000; Santa Cruz Biotechnology, sc-2056) in 5% dry milk solution in TBST for 1 hour at room temperature followed by washing with TBST. Blots were developed using a SuperSignal West Pico chemiluminescent substrate (Thermo Fisher Scientific, Rockford, IL, USA) and processed using x-ray film processor model (BMI no. 122106, Brown’s Medical Imaging, Omaha, NE, USA) or ChemiDoc imaging system (Bio-Rad Laboratories Inc., USA). The representative blots shown were not modified for exposure or contrast from the original images and were not assembled from cropped images.

### Cannulation and viral injection

For stereotaxic cannulation into the NAc, mice were anesthetized with isoflurane (NDC 66794-013-25, Piramal Critical Care, Bethlehem, PA, USA) and placed in a stereotaxic frame (51733U, Stoelting, Wood Dale, IL, USA). The skull was exposed, and a small hole was drilled through the skull and a 26-gauge stainless steel guide cannula was implanted bilaterally above the NAc at the stereotaxic coordinates (anteroposterior (AP): +0.9 mm, mediolateral (ML): ±1 mm, dorsoventral (DV): −4 mm; (*56*), 2001). The guide cannulae were secured to the skull with stainless steel screws (PlasticOne, Roanoke, VA, USA) and dental acrylic cement (B1334, Ortho-Jet, Lang Dental, IL, USA). Experiments were conducted after 10 days of recovery from surgical procedure.

For virus injections, a small hole was drilled above the NAc (AP: +0.9 mm, ML: ±1 mm, DV: −4.2 mm) or PFC (AP: +1.8 mm, ML: ±0.3 mm, DV: −2.3 mm). Virus particles AAV8.GFAP.eGFP and AAV8.GFAP.mCherry-Cre (University of Pennsylvania vector core) were injected (100 nl) using a microliter syringe (NanoFil, World Precision Instruments, Sarasota, FL, USA) with a 33-gauge beveled needle (NF33BV-2, World Precision Instruments). The injection needle was lowered into the NAc core or PFC, and virus particles were delivered at a rate of 1 nl/s using a UMP3 microsyringe pump (World Precision Instruments). The needle was left in place at the injection site for an additional 10 min and thereafter slowly withdrawn over a period of 5 min. Incision was sealed with surgical tissue adhesive (3M Vetbond Tissue Adhesive, MN, USA). Cannula and viral injection locations were verified after the end of behavioral experiments by examining the fixed brain tissue under a light or fluorescent microscope.

### Behavioral analysis

All animals were habituated to the experimental room and handled by the experimenter before the test day. On the test day, animals were placed in the testing room 1 hour before the test. The apparatus was cleaned with 70% ethanol and air-dried in between each animal testing.

### Conditioned place preference

The CPP apparatus consisted of two compartments: black (smooth floor, translucent top) and white (serrated floor, transparent top) chambers each of 20 cm–by–20 cm–by–20 cm dimensions separated by a partition having a guillotine door. After habituation, a pretest was conducted to detect baseline preference for the two compartments: black versus white. During pretest (15 min), the guillotine door was kept open and the time spent by the animal in each chamber was observed and calculated as the preference. We used a biased CPP method in which the preference of the animals to each side of the CPP apparatus was observed in a drug-free state and the drug was paired with the nonpreferred chamber. Animals that spent more than 65% of the total time in any one of the chambers during the pretest were excluded from the study. Conditioning was conducted 1 day after the pretest. The conditioning phase consisted of a total of 10 sessions held on five consecutive days, and each session was run for 30 min. During conditioning, each mouse was confined to one of the compartments by isolating them using a guillotine door. During the first morning session, each animal received cocaine (15 mg/kg, intraperitoneally) in their respective initial nonpreferred compartment. In the second session conducted after 5 hours, vehicle was given in their respective preferred compartment. On the next day, these sessions were reversed, i.e., procedural treatment of the second session of the previous day was carried out during the first session of the next day. This alternating treatment protocol was continued for 5 days of the experiment. Posttest (15 min) was conducted 24 hours after the last conditioning session in a drug-free state. The guillotine door was opened, and each mouse was placed in the apparatus and allowed free access to both the compartments. Time spent (seconds) in each compartment was recorded, and the results were compared with the pretest. The extinction phase consisted of 14 sessions held on seven consecutive days (two sessions per day each of 30 min). The procedure was the same as conditioning; however, the animals were injected with saline in both preferred and nonpreferred chambers. During 7 days of the extinction phase, a 15-min extinction test was carried out on the third (extinction test 1) and fifth (extinction test 2) day to examine the change in preference (time spent in the cocaine-paired chamber) that was recorded while continuing the extinction protocol. Twenty-four hours after the last extinction session, the final extinction test was conducted (extinction test 3). During the reinstatement, a single challenge dose of cocaine was given, and the animals were placed in the CPP chambers. All the CPP sessions were video-recorded and scored using ANY-maze behavioral tracking software (Stoelting Co., IL, USA) for the time spent in each of the two compartments. The time spent in the cocaine-associated chamber during the posttests was analyzed and plotted.

### Diolistic labeling and spine analysis

Diolistic labeling was performed as previously described (*55*, *57*, *58*), with minor modifications. Animals were anesthetized immediately following the CPP session and perfused with 0.1 M PB followed by 1.5% PFA. Brains were removed and postfixed in the same fixative for 1 hour, and coronal slices of 150 μm thickness were prepared. Sections were viewed under a fluorescent microscope to verify viral infection. Tungsten particles (1.3 μm diameter) (Bio-Rad Laboratories, Hercules, CA, USA) coated with the lipophilic carbocyanine dye DiI (Molecular Probes, Life Technologies, Grand Island, NY, USA) were delivered diolistically into the section at 120 psi using a Helios Gene Gun system (Bio-Rad Laboratories, Hercules, CA, USA) with a modified chamber. A polycarbonate filter with 2.0 μm pore size was capped on top of the gun barrel to filter larger particles. DiI was allowed to diffuse along neuron dendrites and axons in PB containing 0.01% (w/v) thimerosal (Sigma-Aldrich, St. Louis, MO, USA) for 24 to 48 hours at 4°C, and then labeled sections were fixed again in 4% PFA for 1 hour. After a brief wash in PB, sections were mounted onto glass slides in Fluoromount-G mounting medium.

Imaging of labeled sections was performed using a Leica TCS SP8 MP scanning confocal microscope (Leica Microsystems). DiI was excited using helium/neon at 543-nm laser line. The entire profile of each DiI-positive neuron to be quantified was acquired using a 63× oil immersion objective. After the neuron was scanned and confirmed as NAc core MSN, its dendrites were focused using a 63× oil immersion objective with ×2 magnification and scanned at 0.1-μm intervals along the *z* axis. The filament module of Imaris software 8.4.1, a 3D imaging software (Bitplane, South Windsor, CT, USA), was used to quantify the spine density and morphology. The minimum end segment diameter (spine head) was set at ≥0.20 μm. We have used a semiautomatic system for the quantification of spines, as described in our previous studies (*55*, *57*). In a semiautomatic system, the spines were manually selected to minimize errors. Semiautomatic quantification of dendritic protrusions and spine classification were performed using Imaris XT. For quantitative analysis, the image was rendered using the surpass module of the Imaris software. Spine quantification commenced on dendrites beginning at 75 μm distal to the soma, and secondary dendrites were preferentially sampled. The minimum length of the dendrite quantified was 25 μm. Counts were made by rotating the segment in 3D to be able to quantify spines in the *z* axis. For each neuron, one or two dendrites were analyzed. For each group, 5 to 10 neurons per animal were analyzed and averaged, and a total of five mice were used for each group. Briefly, parameters for morphological classification of dendritic spines were as follows: mushroom: lengths (spine) < 3 μm and maximum width (head) > mean width (neck) × 2; long and thin: length (spine) > 1 μm and mean width (head) ≥ mean width (neck).

### Statistical analysis

All data are presented as mean ± SEM. Data were analyzed using paired *t* test or unpaired *t* test or one-way or two-way ANOVA with post hoc Bonferroni’s multiple comparisons test. Differences were considered significant if *P* < 0.05. Prism 6 or 7 (GraphPad Software Inc., San Diego, CA, USA) was used for analysis. Data were analyzed by a trained observer blind to the treatment/genotype. The sample sizes for the studies were based on our previous analysis of the role of glutamate receptor in similar CPP paradigm as well as diolistic analysis of spine density and electrophysiology analysis (*57*). We followed the randomization approach based on age and weight for all groups/genotypes used in the present study.

## Supplementary Materials

### This PDF file includes:

Figs. S1 to S6 Table S1 Supplementary Text References

## Notes

View/request a protocol for this paper from *Bio-protocol*.
