# Supplementary material for: Cocaine preference and neuroadaptations are maintained by astrocytic NMDA receptors in the nucleus accumbens
Source: Sci Adv. 2022 Jul 22;8(29):eabo6574. doi: 10.1126/sciadv.abo6574 (PMC9307248; doi:10.1126/sciadv.abo6574)
Supplement: Supplementary file 1 — Figs. S1 to S6 Table S1 Supplementary Text References [file sciadv.abo6574_sm.pdf]

Science Advances
AAAS

Supplementary Materials for

Cocaine preference and neuroadaptations are maintained by astrocytic NMDA receptors in the nucleus accumbens

Gajanan P. Shelkar *et al.*

Corresponding author: Shashank M. Dravid, shashankdravid@creighton.edu;
Gajanan P. Shelkar, gajananshelkar@creighton.edu

Sci. Adv. **8**, eabo6574 (2022)
DOI: 10.1126/sciadv.abo6574

This PDF file includes:

Figs. S1 to S6
Table S1
Supplementary Text
References

Supplementary Materials:

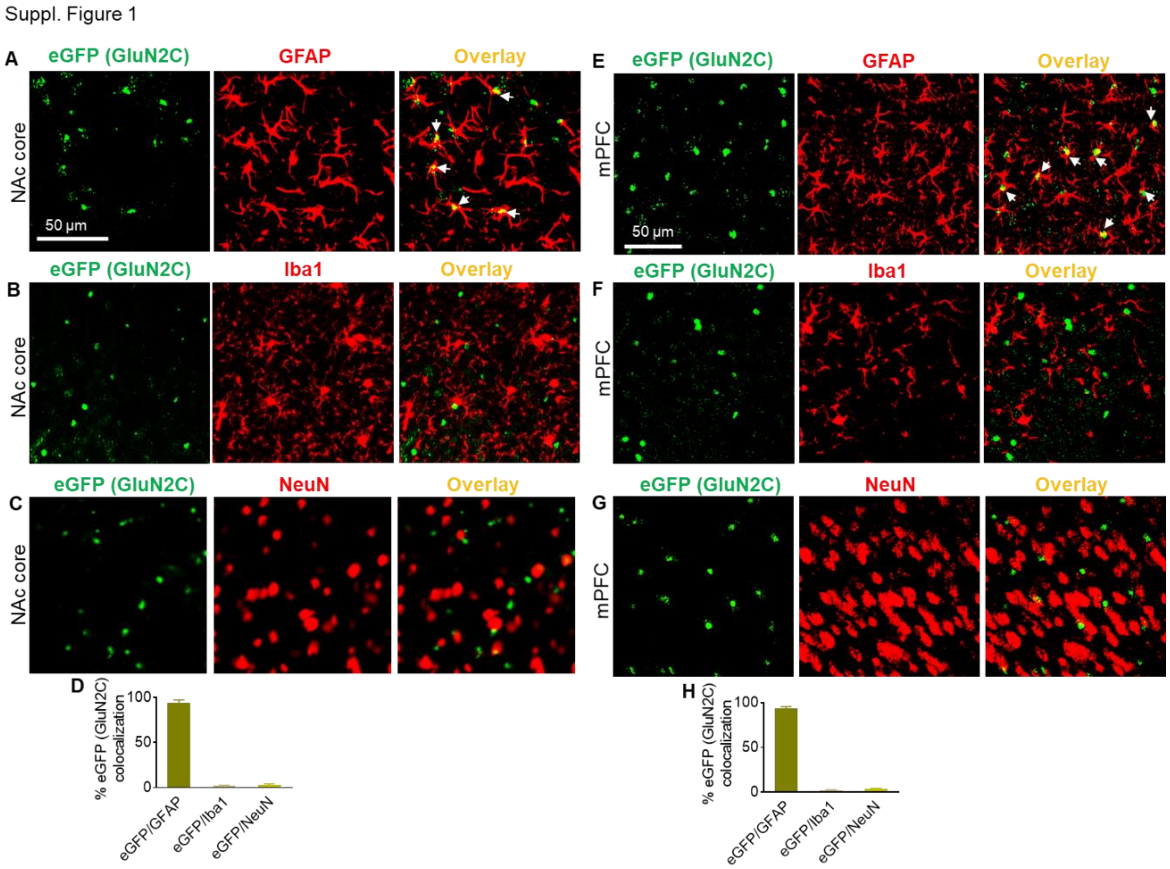

Supplementary Figure 1: GluN2C subunit is specifically expressed in astrocytes but not in neurons in NAc and mPFC. Coronal image from NAc core (**A-C**) or mPFC (**E-G**) of Grin2C$^{tm1(EGFP/cre/ERT2)Wtsi}$ (GluN2C KO) reporter mice immunolabeled for eGFP(GluN2C) + GFAP or eGFP(GluN2C) + NeuN, or eGFP(GluN2C) + Iba1 antibody. Selective colocalization of eGFP with GFAP (~95%) but not with NeuN and Iba1 was observed in both NAc core (**D**) and PFC (**H**), suggesting specific expression of GluN2C in astrocytes.

Suppl. Figure 2

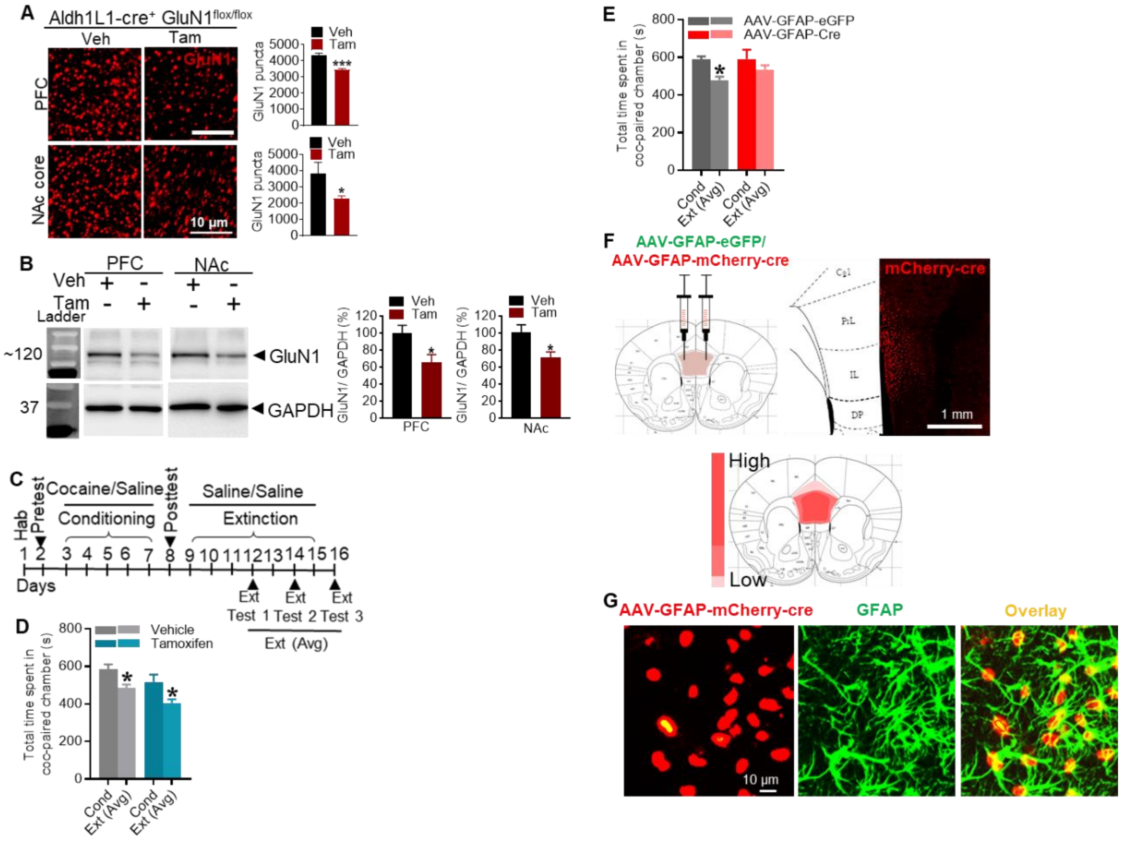

Supplementary Figure 2: Validation of conditional deletion of astrocytic NMDA receptors.

A. Immunohistochemical analysis showing significant reduction in GluN1 puncta in tamoxifen treated mice in the PFC (veh 4360 ± 115.7 vs Tam 3440 ± 84.11; ***$p < 0.0001$, unpaired t-test) and NAc core (veh 3844 ± 675.6 vs tam 2282 ± 162.8; *$p = 0.0483$, unpaired t-test). **B.** Western blot analysis of synaptoneurosomal preparation from PFC and NAc showing reduction in GluN1 expression in tamoxifen treated animals (PFC: veh 100 ± 9.432 vs Tam 65.33 ± 9.598; *$p = 0.05$, and NAc: veh 100.6 ± 9.372 vs tam 70.86 ± 7.11; *$p = 0.0478$, unpaired t-test). **C.** Experimental design used for cocaine conditioning, and extinction experiments. Data from Extinction test 1-3 were collated, averaged and represented as Extinction (Avg) in figs D-E. Hab, habituation; Ext, extinction. **D.** Extinction training significantly reduced time spent in cocaine-paired chamber in

vehicle (*p = 0.0175) and tamoxifen treated Aldh1L1-Cre^{+}GluN1$^{flox/flox}$ mice (*p = 0.0105, two-way ANOVA). **E.** Effect of extinction training on GluN1$^{flox/flox}$ mice injected with AAV-cre and AAV-control into mPFC. Significant reduction in time spent in cocaine-paired chamber was observed in AAV-control-injected mice (*p = 0.0136, two-way ANOVA). Reduction in time spent in cocaine-paired chambers was also seen in AAV-cre injected mice, however it did not reach to significance ($p > 0.05$). **F.** Strategy for local ablation of astrocytic NMDA receptors from mPFC. Schematic showing site of virus injection. Coronal image from GluN1$^{flox/flox}$ mice injected with AAV-GFAP-mCherry-cre showing virus expression in mPFC. The shadowed area (heatmap) in brain schematic showing the extent of overlapping expression patterns across animals in PFC. **G.** Representative images from mPFC of AAV-GFAP-mCherry-cre injected mice immunolabeled for GFAP showing specific colocalization of mCherry-cre with GFAP.

Suppl. Figure 3

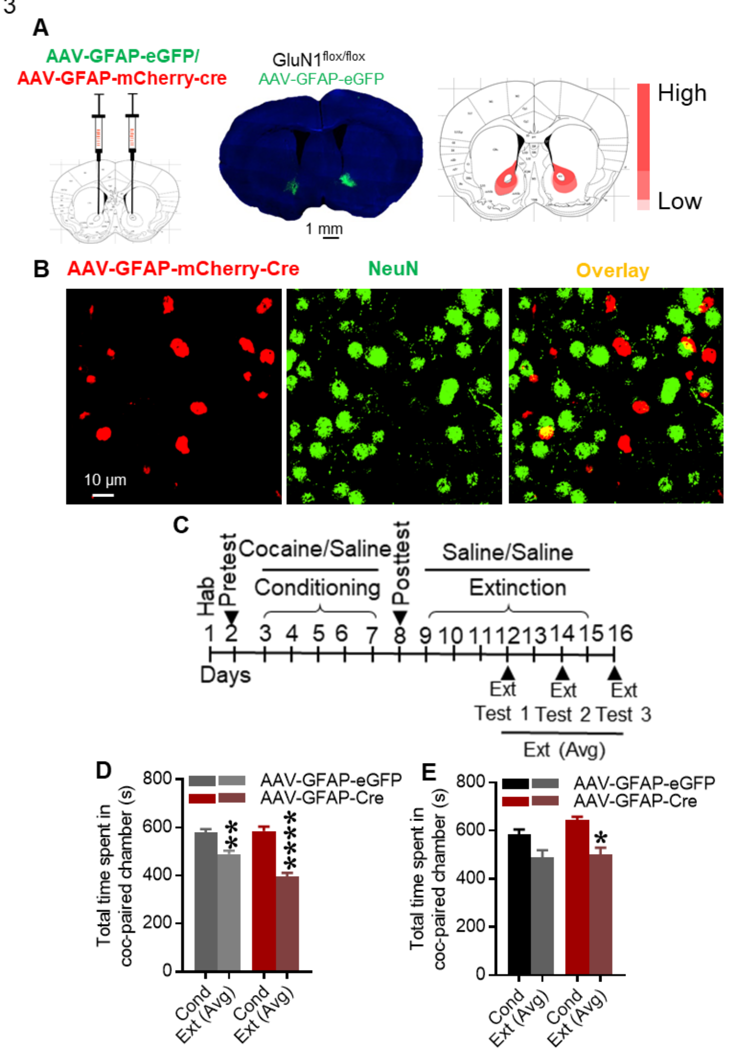

Supplementary Figure 3: Verification of injection sites and specificity of AAV virus. A. Schematic showing the site of virus injections. Coronal image from GluN1$^{flox/flox}$ mice injected with AAV-eGFP showing virus expression in NAc core. The shadowed area (heatmap) in brain schematic showing the extent of overlapping expression patterns across animals in NAc core. **B.** Representative confocal images from NAc core of AAV-GFAP-mCherry-cre injected mice immunolabeled for NeuN antibody. No colocalization of mCherry-cre with NeuN (neuronal marker) was observed. **C.** Experimental design used for cocaine conditioning, and extinction experiments. Data from Extinction test 1-3 were collated, averaged and represented as Extinction

(Avg) in figs D-E. Hab, habituation; Ext, extinction. **D.** Effect of extinction training on GluN1$^{flox/flox}$ mice injected with AAV-cre and AAV-control into NAc core. Significant reduction in time spent in cocaine-paired chamber in both AAV-cre (****p < 0.0001) and AAV-control (**p = 0.0023, one-way ANOVA) injected mice. **E.** Effect of extinction training on WT (no floxed gene) mice injected with AAV-cre and AAV-control into NAc core. Significant reduction in time spent in cocaine-paired chamber in AAV-cre-injected mice (*p < 0.0407, two-way ANOVA).

Suppl. Figure 4

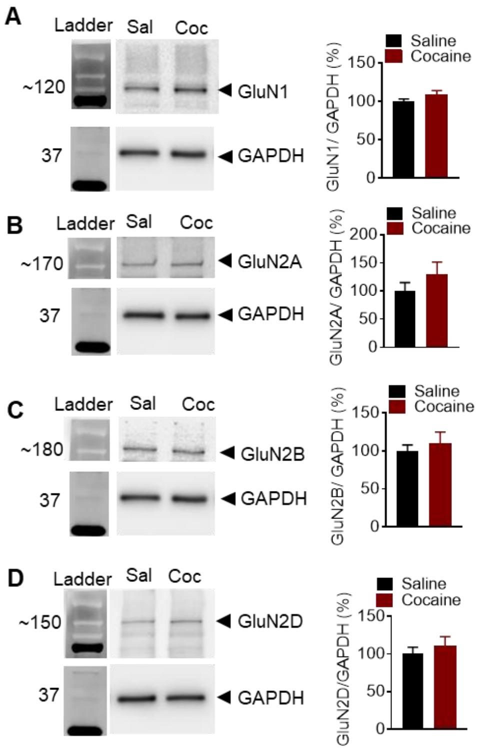

Supplementary Figure 4: Effect of cocaine conditioning on expression of NMDA receptor subunits in NAc. Western blotting analysis of synaptoneurosomal preparation from wildtype mice following saline or cocaine conditioning. No significant change in GluN1 (**A**, p = 0.1497, unpaired t-test, n = 5 each), GluN2A (**B**, p = 0.2978, unpaired t-test, n = 5 each), GluN2B (**C**, p = 0.5823,

unpaired t-test, n = 5 each), GluN2D (**D**, p = 0.4766, unpaired t-test, n = 5 each) was observed following cocaine conditioning compared to saline treatment.

Suppl. Figure 5

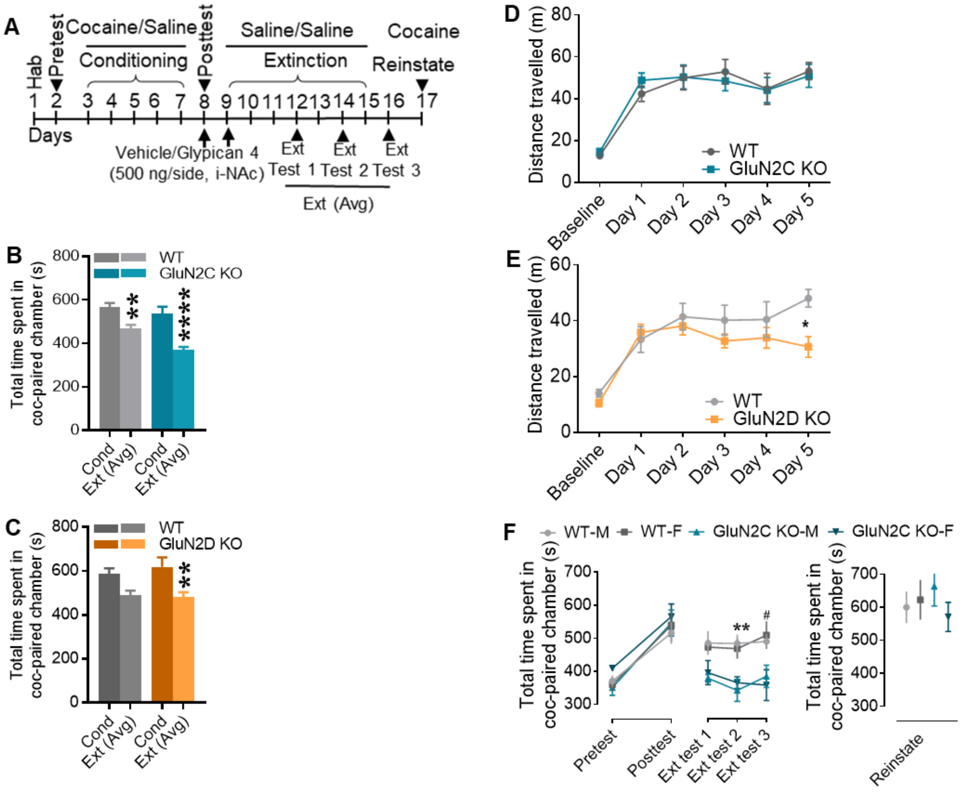

Supplementary Figure 5: Effect of extinction training on cocaine place preference and cocaine-induced locomotion in GluN2C KO and GluN2D KO mice. A. Experimental timeline used for cocaine CPP experiments. Data from Extinction test 1-3 were collated, averaged and represented as Extinction (Avg) in figs B-C. **B.** Extinction training significantly reduced time spent in cocaine-paired chamber in both WT (**p = 0.0015, two-way ANOVA) and GluN2C KO mice (****p < 0.0001, two-way ANOVA). **C.** Extinction training significantly reduced time spent in cocaine-paired chamber in GluN2D KO mice (**p = 0.0028, two-way ANOVA). **D.** Cocaine-induced (15 mg/kg, ip) locomotor activity in WT and GluN2C KO mice. Both WT and GluN2C

KO mice showed similar level of locomotor activity to cocaine treatment. **E.** GluN2D KO mice exhibited significantly reduced (*p = 0.0117, two-way ANOVA) cocaine-induced locomotor activity compared to WT mice. **F.** Sex split analyses for cocaine conditioning, extinction and reinstatement in WT and GluN2C KO mice. No significant difference in between the sexes in WT and GluN2C KO following cocaine conditioning (WT-M vs WT-F: $p > 0.999$; GluN2C KO-M vs GluN2C-KO-F: $p > 0.999$, two-way ANOVA), and extinction (WT-M vs WT-F: $p > 0.999$, GluN2C-M vs GluN2C-F: $p > 0.999$ at ext. 1, 2 and 3). Both GluN2C KO male and female mice exhibit enhanced extinction with lower time spent in cocaine-paired chamber compared to respective WT (GluN2C KO-M, **p = 0.0068, at ext. test 2, and GluN2C KO-F, #p = 0.0115 at ext. test 3). Two-way ANOVA showed significant effect of genotype on extinction [$F (3, 61) = 13.23$, $p < 0.0001$; n = 8 WT-M, 6 WT-F, 6 each GluN2C KO-M and F]. No sex specific differences in reinstatement were observed between the sexes in WT and GluN2C KO mice ($p = 0.6730$, one-way ANOVA).

Suppl. Figure 6

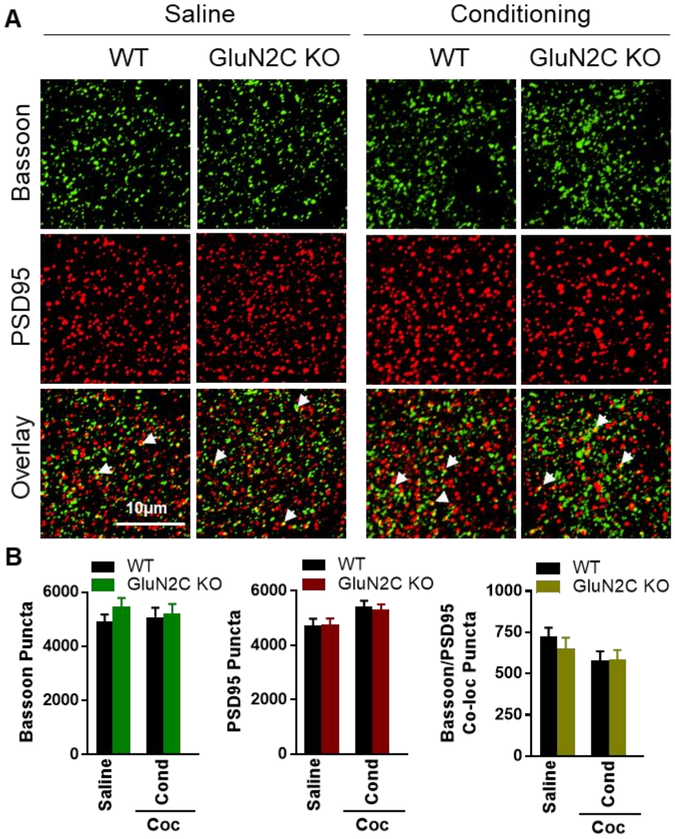

Supplementary Figure 6: No change in bassoon-PSD95 colocalized puncta in WT and GluN2C KO mice following cocaine conditioning. A. Representative confocal images from NAc core from WT and GluN2C KO mice immunostained for bassoon and PSD95 after saline and cocaine conditioning **B.** Quantification of immunostaining; cocaine conditioning in WT mice did not produce any significant changes in bassoon, PSD95 or bassoon/PSD95-colocalized puncta ($p > 0.9999$ each). No significant differences were observed in bassoon, PSD95 or bassoon/PSD95-colocalized puncta in WT compared to GluN2C KO mice ($p > 0.9999$ each).

Supplementary table 1: Data representing the change in time spent in cocaine-paired chamber

			Change in time spent in cocaine-paired side (s)			
			Conditioning (Posttest-Pretest)	Ext test 1 (Posttest-Ext test 1)	Ext test 2 (Posttest-Ext test 2)	Ext test 3 (Posttest-Ext test 3)
Figure 2 E	AldhGluN1Flox+/+	Vehicle	290.7 ± 26.8	81.33 ±40.37	75.66±28.42	38.26±32.23
		Tamoxifen	236 ± 29.24	67.1±34.76	112.02±29.23	168.1±34.79
Figure 2 G	GluN1Flox+/+	AAV-Control	208.3 ± 14.88	78.39 ±33.51	113.09±36.40	116.34±25.89
		AAV-Cre	245 ± 54.07	72.52±66.90	99.68±46.52	89.03±30.05
Figure 3 E	GluN1Flox+/+	AAV-Control	215.2 ± 24.94	104.24 ±16.99	91.5±26.139	97.71±27.16
		AAV-Cre	226.7 ± 22.81	163.78±32.66	182.04±28.97	209.12±26.14
Figure 3 G	WT	AAV-Control	250.5 ± 58.36	97.6±57.44	76.94±49.51	116.26±68.52
		AAV-Cre	270.4 ± 29.71	137.62±60.55	111.52±57.84	124.33±64.27
Figure 5 C	WT	Vehicle	181.7 ± 41.69	8.52±77.51	3.18±61.48	
		DQP	227.9 ± 51.4	16.62±51.95	279.7±80.73	
Figure 5 F	WT		180.7 ± 26.13	45.57±31.67	46.55±27.62	19.87±25.45
	GluN2C KO		185.8 ± 32.36	229.62±38.53	232.55±39.64	201.94±45.8
Figure 5 G	WT		231.8 ± 32.94	63.45±35.36	121.06±38.56	104.05±41.42
	GluN2D KO		262.2 ± 56.23	125.86±70.82	156.72±80.46	127.86±64.17
Figure 8 E	WT	Vehicle	244.8 ± 41.03	124.43±48.88	187.78±50.46	148.73±76.68
		Glypican 4	278.7 ± 44.57	90.26±43.15	160.825±46.33	119.27±75.75
Figure 8 F	GluN2C KO	Vehicle	177.8 ± 45.23	215.1±42.36	222.86±32.14	176.39±36.22
		Glypican 4	228.2 ± 50.4	63.38±81.48	80.88±75.02	66.98±50.57

Statistical information:

The sample sizes for the studies were based on our previous analysis of role of glutamate receptor in similar CPP paradigm as well as diolistic analysis of spine density and electrophysiology analysis *(58)*. A power analysis (alpha = 0.05, power = 0.9) using standard deviation estimates and mean difference in behavioral end points suggested that a sample size of 5-8 was needed. The sample size for behavioral experiments and IHC studies, were consistent with the guidance for experimental design and analysis to permit sufficient statistical analysis *(59)*. We also followed randomization approach based on age and weight for all groups/genotypes used in the present study. Additional information on the exact number of animals used in each experiment is included in the figure legend of respective figure. All experiments were replicated at least 2-4 times in the laboratory. All data describe biological replicates.

REFERENCES AND NOTES

1. P. W. Kalivas, N. D. Volkow, The neural basis of addiction: A pathology of motivation and choice. *Am. J. Psychiatry* **162**, 1403–1413 (2005).

2. J. T. Gass, L. J. Chandler, The plasticity of extinction: Contribution of the prefrontal cortex in treating addiction through inhibitory learning. *Front. Psychiatry* **4**, 46 (2013).

3. M. Davis, M. Barad, M. Otto, S. Southwick, Combining pharmacotherapy with cognitive behavioral therapy: Traditional and new approaches. *J. Trauma. Stress* **19**, 571–581 (2006).

4. M. E. Bouton, Context and behavioral processes in extinction. *Learn. Mem.* **11**, 485–494 (2004).

5. K. Moussawi, W. Zhou, H. Shen, C. M. Reichel, R. E. See, D. B. Carr, P. W. Kalivas, Reversing cocaine-induced synaptic potentiation provides enduring protection from relapse. *Proc. Natl. Acad. Sci. U.S.A.* **108**, 385–390 (2011).

6. M. D. Scofield, J. A. Heinsbroek, C. D. Gipson, Y. M. Kupchik, S. Spencer, A. C. W. Smith, D. Roberts-Wolfe, P. W. Kalivas, The nucleus accumbens: Mechanisms of addiction across drug classes reflect the importance of glutamate homeostasis. *Pharmacol. Rev.* **68**, 816–871 (2016).

7. M. D. Scofield, P. W. Kalivas, Astrocytic dysfunction and addiction: Consequences of impaired glutamate homeostasis. *Neuroscientist* **20**, 610–622 (2014).

8. N. J. Allen, C. Eroglu, Cell biology of astrocyte-synapse interactions. *Neuron* **96**, 697–708 (2017).

9. M. D. Scofield, H. A. Boger, R. J. Smith, H. Li, P. G. Haydon, P. W. Kalivas, Gq-DREADD selectively initiates glial glutamate release and inhibits cue-induced cocaine seeking. *Biol. Psychiatry* **78**, 441–451 (2015).

10. M. D. Scofield, H. Li, B. M. Siemsen, K. L. Healey, P. K. Tran, N. Woronoff, H. A. Boger, P. W. Kalivas, K. J. Reissner, Cocaine self-administration and extinction leads to reduced glial fibrillary acidic protein expression and morphometric features of astrocytes in the nucleus accumbens core. *Biol. Psychiatry* **80**, 207–215 (2016).

11. E. C. Dumont, G. P. Mark, S. Mader, J. T. Williams, Self-administration enhances excitatory synaptic transmission in the bed nucleus of the stria terminalis. *Nat. Neurosci.* **8**, 413–414 (2005).

12. S. Kourrich, P. E. Rothwell, J. R. Klug, M. J. Thomas, Cocaine experience controls bidirectional synaptic plasticity in the nucleus accumbens. *J. Neurosci.* **27**, 7921–7928 (2007).

13. B. R. Lee, Y. Dong, Cocaine-induced metaplasticity in the nucleus accumbens: Silent synapse and beyond. *Neuropharmacology* **61**, 1060–1069 (2011).

14. U. Lalo, Y. Pankratov, F. Kirchhoff, R. A. North, A. Verkhratsky, NMDA receptors mediate neuron-to-glia signaling in mouse cortical astrocytes. *J. Neurosci.* **26**, 2673–2683 (2006).

15. O. Palygin, U. Lalo, Y. Pankratov, Distinct pharmacological and functional properties of NMDA receptors in mouse cortical astrocytes. *Br. J. Pharmacol.* **163**, 1755–1766 (2011).

16. A. Ravikrishnan, P. J. Gandhi, G. P. Shelkar, J. Liu, R. Pavuluri, S. M. Dravid, Region-specific expression of NMDA receptor GluN2C subunit in parvalbumin-positive neurons and astrocytes: Analysis of GluN2C expression using a novel reporter model. *Neuroscience* **380**, 49–62 (2018).

17. H. A. Alsaad, N. W. DeKorver, Z. Mao, S. M. Dravid, J. Arikkath, D. T. Monaghan, In the telencephalon, GluN2C NMDA receptor subunit mRNA is predominately expressed in glial cells and GluN2D mRNA in interneurons. *Neurochem. Res.* **44**, 61–77 (2019).

18. J. D. Cahoy, B. Emery, A. Kaushal, L. C. Foo, J. L. Zamanian, K. S. Christopherson, Y. Xing, J. L. Lubischer, P. A. Krieg, S. A. Krupenko, W. J. Thompson, B. A. Barres, A transcriptome database for astrocytes, neurons, and oligodendrocytes: A new resource for understanding brain development and function. *J. Neurosci.* **28**, 264–278 (2008).

19. I. Karavanova, K. Vasudevan, J. Cheng, A. Buonanno, Novel regional and developmental NMDA receptor expression patterns uncovered in NR2C subunit-beta-galactosidase knock-in mice. *Mol. Cell. Neurosci.* **34**, 468–480 (2007).

20. Y. Zhang, K. Chen, S. A. Sloan, M. L. Bennett, A. R. Scholze, S. O'Keeffe, H. P. Phatnani, P. Guarnieri, C. Caneda, N. Ruderisch, S. Deng, S. A. Liddelow, C. Zhang, R. Daneman, T. Maniatis, B.

A. Barres, J. Q. Wu, An RNA-sequencing transcriptome and splicing database of glia, neurons, and vascular cells of the cerebral cortex. *J. Neurosci.* **34**, 11929–11947 (2014).

21. J. Peters, R. T. LaLumiere, P. W. Kalivas, Infralimbic prefrontal cortex is responsible for inhibiting cocaine seeking in extinguished rats. *J. Neurosci.* **28**, 6046–6053 (2008).

22. B. L. Warren, L. Kane, M. Venniro, P. Selvam, R. Quintana-Feliciano, M. P. Mendoza, R. Madangopal, L. Komer, L. R. Whitaker, F. J. Rubio, J. M. Bossert, D. Caprioli, Y. Shaham, B. T. Hope, Separate vmPFC ensembles control cocaine self-administration versus extinction in rats. *J. Neurosci.* **39**, 7394–7407 (2019).

23. Y. Dong, E. J. Nestler, The neural rejuvenation hypothesis of cocaine addiction. *Trends Pharmacol. Sci.* **35**, 374–383 (2014).

24. H. W. Shen, S. Toda, K. Moussawi, A. Bouknight, D. S. Zahm, P. W. Kalivas, Altered dendritic spine plasticity in cocaine-withdrawn rats. *J. Neurosci.* **29**, 2876–2884 (2009).

25. K. W. Lee, Y. Kim, A. M. Kim, K. Helmin, A. C. Nairn, P. Greengard, Cocaine-induced dendritic spine formation in D1 and D2 dopamine receptor-containing medium spiny neurons in nucleus accumbens. *Proc. Natl. Acad. Sci. U.S.A.* **103**, 3399–3404 (2006).

26. J. Wang, L. M. Holt, H. H. Huang, S. R. Sesack, E. J. Nestler, Y. Dong, Astrocytes in cocaine addiction and beyond. *Mol. Psychiatry* **27**, 652–668 (2022).

27. J. Wang, K. L. Li, A. Shukla, A. Beroun, M. Ishikawa, X. Huang, Y. Wang, Y. Q. Wang, Y. Yang, N. D. Bastola, H. H. Huang, L. E. Kramer, T. Chao, Y. H. Huang, S. R. Sesack, E. J. Nestler, O. M. Schlüter, Y. Dong, Cocaine triggers astrocyte-mediated synaptogenesis. *Biol. Psychiatry* **89**, 386–397 (2021).

28. C. Luscher, R. C. Malenka, Drug-evoked synaptic plasticity in addiction: From molecular changes to circuit remodeling. *Neuron* **69**, 650–663 (2011).

29. C. Bats, L. Groc, D. Choquet, The interaction between Stargazin and PSD-95 regulates AMPA receptor surface trafficking. *Neuron* **53**, 719–734 (2007).

30. I. Farhy-Tselnicker, A. C. M. van Casteren, A. Lee, V. T. Chang, A. R. Aricescu, N. J. Allen, Astrocyte-secreted glypican 4 regulates release of neuronal pentraxin 1 from axons to induce functional synapse formation. *Neuron* **96**, 428–445.e13 (2017).

31. S. Bhattacharya, A. Khatri, S. A. Swanger, J. O. Di Raddo, F. Yi, K. B. Hansen, H. Yuan, S. F. Traynelis, Triheteromeric GluN1/GluN2A/GluN2C NMDARs with unique single-channel properties are the dominant receptor population in cerebellar granule cells. *Neuron* **99**, 315–328.e5 (2018).

32. M. E. Joffe, B. A. Grueter, Cocaine experience enhances thalamo-accumbens N-methyl-D-aspartate receptor function. *Biol. Psychiatry* **80**, 671–681 (2016).

33. A. Kruyer, P. W. Kalivas, Astrocytes as cellular mediators of cue reactivity in addiction. *Curr. Opin. Pharmacol.* **56**, 1–6 (2021).

34. M. D. Scofield, Exploring the role of astroglial glutamate release and association with synapses in neuronal function and behavior. *Biol. Psychiatry* **84**, 778–786 (2018).

35. H. Chai, B. Diaz-Castro, E. Shigetomi, E. Monte, J. C. Octeau, X. Yu, W. Cohn, P. S. Rajendran, T. M. Vondriska, J. P. Whitelegge, G. Coppola, B. S. Khakh, Neural circuit-apecialized astrocytes: Transcriptomic, proteomic, morphological, and functional evidence. *Neuron* **95**, 531–549.e9 (2017).

36. A. Testen, M. T. Sepulveda-Orengo, C. H. Gaines, K. J. Reissner, Region-specific reductions in morphometric properties and synaptic colocalization of astrocytes following cocaine self-administration and extinction. *Front. Cell. Neurosci.* **12**, 246 (2018).

37. S. A. Carmack, J. S. Kim, J. R. Sage, A. W. Thomas, K. N. Skillicorn, S. G. Anagnostaras, The competitive NMDA receptor antagonist CPP disrupts cocaine-induced conditioned place preference, but spares behavioral sensitization. *Behav. Brain Res.* **239**, 155–163 (2013).

38. P. Hyytia, P. Backstrom, S. Liljequist, Site-specific NMDA receptor antagonists produce differential effects on cocaine self-administration in rats. *Eur. J. Pharmacol.* **378**, 9–16 (1999).

39. P. Backstrom, P. Hyytia, Involvement of AMPA/kainate, NMDA, and mGlu5 receptors in the nucleus accumbens core in cue-induced reinstatement of cocaine seeking in rats. *Psychopharmacology* **192**, 571–580 (2007).

40. C. L. Heusner, R. D. Palmiter, Expression of mutant NMDA receptors in dopamine D1 receptor-containing cells prevents cocaine sensitization and decreases cocaine preference. *J. Neurosci.* **25**, 6651–6657 (2005).

41. M. Sikora, K. Tokarski, B. Bobula, J. Zajdel, K. Jastrzębska, P. E. Cieślak, M. Zygmunt, J. Sowa, M. Smutek, K. Kamińska, K. Gołembiowska, D. Engblom, G. Hess, R. Przewlocki, J. R. Parkitna, NMDA receptors on dopaminoceptive neurons are essential for drug-induced conditioned place preference. *eNeuro* **3**, ENEURO.0084-15.2016 (2016).

42. S. F. Traynelis, L. P. Wollmuth, C. J. McBain, F. S. Menniti, K. M. Vance, K. K. Ogden, K. B. Hansen, H. Yuan, S. J. Myers, R. Dingledine, Glutamate receptor ion channels: Structure, regulation, and function. *Pharmacol. Rev.* **62**, 405–496 (2010).

43. T. E. Brown, B. R. Lee, P. Mu, D. Ferguson, D. Dietz, Y. N. Ohnishi, Y. Lin, A. Suska, M. Ishikawa, Y. H. Huang, H. Shen, P. W. Kalivas, B. A. Sorg, R. S. Zukin, E. J. Nestler, Y. Dong, O. M. Schluter, A silent synapse-based mechanism for cocaine-induced locomotor sensitization. *J. Neurosci.* **31**, 8163–8174 (2011).

44. S. Jones, A. J. Gibb, Functional NR2B- and NR2D-containing NMDA receptor channels in rat substantia nigra dopaminergic neurones. *J. Physiol.* **569**, 209–221 (2005).

45. A. C. Boudreau, C. R. Ferrario, M. J. Glucksman, M. E. Wolf, Signaling pathway adaptations and novel protein kinase A substrates related to behavioral sensitization to cocaine. *J. Neurochem.* **110**, 363–377 (2009).

46. M. E. Wolf, C. R. Ferrario, AMPA receptor plasticity in the nucleus accumbens after repeated exposure to cocaine. *Neurosci. Biobehav. Rev.* **35**, 185–211 (2010).

47. N. Matsuo, L. Reijmers, M. Mayford, Spine-type-specific recruitment of newly synthesized AMPA receptors with learning. *Science* **319**, 1104–1107 (2008).

48. N. J. Allen, M. L. Bennett, L. C. Foo, G. X. Wang, C. Chakraborty, S. J. Smith, B. A. Barres, Astrocyte glypicans 4 and 6 promote formation of excitatory synapses via GluA1 AMPA receptors. *Nature* **486**, 410–414 (2012).

49. K. F. Tolias, J. B. Bikoff, A. Burette, S. Paradis, D. Harrar, S. Tavazoie, R. J. Weinberg, M. E. Greenberg, The Rac1-GEF Tiam1 couples the NMDA receptor to the activity-dependent development of dendritic arbors and spines. *Neuron* **45**, 525–538 (2005).

50. J. Liu, G. P. Shelkar, F. Zhao, R. P. Clausen, S. M. Dravid, Modulation of burst firing of neurons in nucleus reticularis of the thalamus by GluN2C-containing NMDA receptors. *Mol. Pharmacol.* **96**, 193–203 (2019).

51. P. Mullasseril, K. B. Hansen, K. M. Vance, K. K. Ogden, H. Yuan, N. L. Kurtkaya, R. Santangelo, A. G. Orr, P. le, K. M. Vellano, D. C. Liotta, S. F. Traynelis, A subunit-selective potentiator of NR2C- and NR2D-containing NMDA receptors. *Nat. Commun.* **1**, 90 (2010).

52. M. Jessen, K. Frederiksen, F. Yi, R. P. Clausen, K. B. Hansen, H. Bräuner-Osborne, P. Kilburn, A. Damholt, Identification of AICP as a GluN2C-selective N-methyl-d-aspartate receptor superagonist at the GluN1 glycine site. *Mol. Pharmacol.* **92**, 151–161 (2017).

53. G. P. Shelkar, R. Pavuluri, P. J. Gandhi, A. Ravikrishnan, D. Y. Gawande, J. Liu, D. J. Stairs, R. R. Ugale, S. M. Dravid, Differential effect of NMDA receptor GluN2C and GluN2D subunit ablation on behavior and channel blocker-induced schizophrenia phenotypes. *Sci. Rep.* **9**, 7572-019 (2019).

54. J. Liu, G. P. Shelkar, P. J. Gandhi, D. Y. Gawande, A. Hoover, R. M. Villalba, R. Pavuluri, Y. Smith, S. M. Dravid, Striatal glutamate delta-1 receptor regulates behavioral flexibility and thalamostriatal connectivity. *Neurobiol. Dis.* **137**, 104746 (2020).

55. S. C. Gupta, R. Yadav, R. Pavuluri, B. J. Morley, D. J. Stairs, S. M. Dravid, Essential role of GluD1 in dendritic spine development and GluN2B to GluN2A NMDAR subunit switch in the cortex and hippocampus reveals ability of GluN2B inhibition in correcting hyperconnectivity. *Neuropharmacology* **93**, 274–284 (2015).

56. G. Paxinos, K. B. J. Franklin, The mouse brain in stereotaxic coordinates (San Diego, CA: Academic Press, 2001).

57. J. Liu, P. J. Gandhi, R. Pavuluri, G. P. Shelkar, S. M. Dravid, Glutamate delta-1 receptor regulates cocaine-induced plasticity in the nucleus accumbens. *Transl. Psychiatry* **8**, 219 (2018).

58. S. C. Gupta, A. Ravikrishnan, J. Liu, Z. Mao, R. Pavuluri, B. G. Hillman, P. J. Gandhi, D. J. Stairs, M. Li, R. R. Ugale, D. T. Monaghan, S. M. Dravid, The NMDA receptor GluN2C subunit controls cortical excitatory-inhibitory balance, neuronal oscillations and cognitive function. *Sci. Rep.* **6**, 38321 (2016).

59. M. J. Curtis, S. Alexander, G. Cirino, J. R. Docherty, C. H. George, M. A. Giembycz, D. Hoyer, P. A. Insel, A. A. Izzo, Y. Ji, D. J. MacEwan, C. G. Sobey, S. C. Stanford, M. M. Teixeira, S. Wonnacott, A. Ahluwalia, Experimental design and analysis and their reporting II: Updated and simplified guidance for authors and peer reviewers. *Br. J. Pharmacol.* **175**, 987–993 (2018).
